# Epinephrine Improves the Efficacy of Nebulized Hypertonic Saline in Moderate Bronchiolitis: A Randomised Clinical Trial

**DOI:** 10.1371/journal.pone.0142847

**Published:** 2015-11-17

**Authors:** J. Carlos Flores-González, Miguel A. Matamala-Morillo, Patricia Rodríguez-Campoy, Juan J. Pérez-Guerrero, Belén Serrano-Moyano, Paloma Comino-Vazquez, Encarnación Palma-Zambrano, Rocio Bulo-Concellón, Vanessa Santos-Sánchez, Alfonso M. Lechuga-Sancho, Bronchiolitis of Cadiz Study group (BronCaS)

**Affiliations:** 1 Department of Clinical Pediatrics, Hospital Universitario Puerta del Mar, Cádiz, Spain; 2 Department of Pharmacy, Hospital Universitario Puerta del Mar, Cádiz, Spain; 3 Foundation for Biomedical Research Management, Cádiz, Spain; 4 Department of Maternal and Child Health Care and Radiology, University of Cádiz, Cádiz, Spain; Fondazione IRCCS Ca' Granda Ospedale Maggiore Policlinico, Università degli Studi di Milano, ITALY

## Abstract

**Background and Aims:**

There is no evidence that the epinephrine-3% hypertonic saline combination is more effective than 3% hypertonic saline alone for treating infants hospitalized with acute bronchiolitis. We evaluated the efficacy of nebulized epinephrine in 3% hypertonic saline.

**Patients and Methods:**

We performed a randomized, double-blind, placebo-controlled clinical trial in 208 infants hospitalized with acute moderate bronchiolitis. Infants were randomly assigned to receive nebulized 3% hypertonic saline with either 3 mL of epinephrine or 3 mL of placebo, administered every four hours. The primary outcome measure was the length of hospital stay.

**Results:**

A total of 185 infants were analyzed: 94 in the epinephrine plus 3% hypertonic saline group and 91 in the placebo plus 3% hypertonic saline group. Baseline demographic and clinical characteristics were similar in both groups. Length of hospital stay was significantly reduced in the epinephrine group as compared with the placebo group (3.94 ±1.88 days vs. 4.82 ±2.30 days, P = 0.011). Disease severity also decreased significantly earlier in the epinephrine group (P = 0.029 and P = 0.036 on days 3 and 5, respectively).

**Conclusions:**

In our setting, nebulized epinephrine in 3% hypertonic saline significantly shortens hospital stay in hospitalized infants with acute moderate bronchiolitis compared to 3% hypertonic saline alone, and improves the clinical scores of severity from the third day of treatment, but not before.

**Trial Registration:**

EudraCT 2009-016042-57

## Introduction

Bronchiolitis is the most frequent lower respiratory tract infection in the first year of life and one of the main reasons for hospitalization in this age group [1]. Between 1% and 3.5% of otherwise healthy infants, are hospitalized for bronchiolitis every year [2–5].The most common cause of this respiratory infection is the respiratory syncytial virus (RSV) [6]. Pathophysiologically, acute bronchiolitis is characterized by an obstruction of the small airways due to peribronchial mononuclear cell infiltration, increased mucus production, mucosal and submucosal edema, and necrosis and sloughing of epithelial cells [7].

Nebulized 3% hypertonic saline, may be helpful in the treatment of infants with acute bronchiolitis, since it lowers the viscosity and elasticity of mucus by breaking ionic bonds, it rehydrates secretions by inducing osmotic flow, it also stimulates cilial beat by releasing prostaglandin E_2_, and finally nebulized 3% hypertonic saline favors the clearing of mucus by inducing cough [8–11]. In fact, the most recent Clinical Practice Guidelines recommends the use of hypertonic saline rather than physiologic saline [12;13]. This is though, a weak recommendation due to inconsistent findings, that have been challenged by very recent data [14].

There is not enough evidence of the benefit of any given bronchodilator in the treatment of acute bronchiolitis to support recommending its use in the mentioned Guidelines [12] since no bronchodilator have provided any clinical benefit compared with normal saline [15–20]. As mucosal edema is the main cause of bronchiolar obstruction, it has been postulated that epinephrine might be the best treatment option because of its α-adrenergic properties on bronchiolar mucosae [7]. However, studies comparing the efficacy of nebulized salbutamol versus nebulized epinephrine in normal saline, have shown that while epinephrine may transiently improve respiratory distress, it fails to shorten the length of hospital stay [16;21–25]. To date, epineprhine’s efficacy in bronchiolitis has only been especifically tested diluted in normal saline, not in 3% saline.

Recently, 3% hypertonic saline with albuterol obtained a minimal reduction in hospital stay, when compared with 0.9% saline with albuterol (3.16 days versus 3.92), though the difference did not reach statistical significance [26].

To the best of our knowledge, there has not been yet a clinical trial specifically designed to if epinephrine would make a difference in the length of hospital stay between patients receiving 3% saline nebulizations, or at least, to test if it induces a decrease the relative risk of prolonged hospital stay (i.e. more than 4 days). We conducted a double-blind, randomized, placebo-controlled trial, to compare the efficacy of nebulized epinephrine in 3% hypertonic saline versus nebulized placebo in 3% hypertonic saline. The primary efficacy outcome was the reduction in length of hospital stay in infants with acute moderate bronchiolitis.

## Patients and Methods

### Patients

We conducted a randomized, double-blind, placebo-controlled clinical trial. Eligible patients included infants aged under 24 months admitted to Hospital Universitario Puerta del Mar in Cádiz, Spain, between October 2011 and May 2014 with a clinical diagnosis of acute bronchiolitis (International Diseases Classification, Ninth revision, code 466.1), classified as moderate in severity. The diagnosis was based on a first episode of respiratory distress with wheezing and/or crackles, preceded by an infection of the upper airways. Disease severity was evaluated using the Wood-Downes clinical scoring system modified by Ferres (WDF)[27], which defines as moderate intensity, the clinical cases scoring between 4 and 7 (Table 1). Infants were excluded if they had any of the following risk factors: premature birth as defined by the World Health Organization (< 37 weeks), in infants with an adjusted age of less than 6 weeks at the time of enrollment, chronic respiratory disease, hemodynamically significant heart disease, immunodeficiency, and neuromuscular disease. Infants with previous episodes of wheezing or a physician’s diagnosis of asthma were also excluded. Finally, we also excluded patients receiving other non-study treatments during hospitalization.

**Table 1:** Wood-Downes Clinical Scoring System Modified by Ferres.

	0	1	2	3
Wheezing	None	End expiration	Entire expiratory phase	Inspiration and expiration
Retractions	None	Subcostal or lower intercostal	1 + supraclavicular + nasal flaring	2 + suprasternal + lower intercostal
Respiratory rate—breaths/min	< 30	31–45	46–60	>60
Heart rate—beats/min	< 120	> 120		
Inspiratory breath sounds	Normal	Regular, symmetrical	Markedly silent, symmetrical	Silent thorax, no wheezing
Cyanosis	Not present	Present		

A score of 1–3 points denotes mild bronchiolitis; 4–7 moderate bronchiolitis; and 8–14 ssevere bronchiolitis.

Every infant evaluated for clinically suspected bronchiolitis, underwent a full physical examination (including the calculation of WDF score), and a medical history was obtained from the parents or legal representatives. When infants were eligible, the treating physician would offer the parents or legal guardians to voluntarily participate in the study and informed consent was obtained in all cases.

Once included, the following data were recorded on admission as per research protocol, by a research collaborator: age, sex, family history of atopy, parental smoking, personal history of atopy, personal history of cow’s milk protein allergy, type of feeding (breastfeeding vs. formula feed or both), number of siblings, attendance or not at a nursery school, WDF score, and current medication (number of days taking inhaled salbutamol or oral corticosteroids). Infants with oxygen saturation of 94% or below received oxygen supplementation via a nasal cannula with the lowest possible fraction of inspired oxygen (Fio_2_) to achieve a saturation level of above 94%.

### Intervention

Infants were randomized to one of two groups by a computer-generated random sequence, using the creative commons licensed software available at www.randomisatiom.com. 216 subjects were randomized into 27 blocks of 8 patients. The hospital pharmacy department labeled the treatment solutions with a code to mask doctors, investigators, and patients until the last patient recruited was discharged.

In the first group, patients received nebulized epinephphine (3 ml of a 1:1000 solution), in 3% hypertonic saline (7 mL) and in the second group, they received nebulized 3% hypertonic saline (7 mL) plus 3 mL placebo (sterile water). The solutions were administered initially every 4 hours. Both solutions were identical in color, smell, consistency, volume and final sodium concentration. Patients would drop out of the study if after inclusion their legal representatives withdrew consent or received any other non-study medications.

Treating physicians followed our institutions’ protocol for the treatment of bronchiolitis, adopted form the 2006 guidelines [1]. Thus, infants received the same standard support (elevation of the head of bed, supplemental oxygen when oxygen saturation dropped below 94%, acetaminophen if fever, and a nasal lavage with sterile saline before and after the administration of the nebulized solution). Their diet was prescribed by their treating physician according to the degree of respiratory distress and oral tolerance. Every patient was monitored with a pulse oxymeter (Nellcor, Oximax N-600x) until oxygen saturation stabilized above 94% without any supplemental oxygen. The nebulized solution was administered by means of a mask using an ultrasonic hospital nebulizer (Shinmed model Sw918) with a frequency of 1.7 MHz and a mist particle size of 1 to 5 μm.

### Assessments and efficacy outcome

The primary efficacy outcome was length of hospital stay (LOS), defined as the number of days from admission to the time at which the patient fulfilled the study discharge criteria: a WDF score of 3 or less, an oxygen saturation of 97% or more without supplemental oxygen, adequate oral tolerance, and no further need for nebulized therapy.

Secondary efficacy variables (respiratory rate, heart rate, oxygen saturation, inhaled Fio_2_, WDF score, and nebulization intervals (hours) were also recorded by research collaborators. The respiratory rate was measured over a period of 1 minute. Note was also taken of any adverse event observed during hospitalization (tachycardia, sweating, pallor, trembling, hypertension) and of the need for intensive care.

### Study oversight

The study was approved by the Puerta del Mar University Hospital’s research ethics committee and the Spanish Agency for Medicines and Medical Devices. Informed consent was signed by the parents or legal representatives of every infant who participated in the study. This study is in accordance with the CONSORT 2010 (S1 File: consort 2010 Compliance Checklist). Full version of the study protocol is available as supporting information (S2 File: Study Protocol).

### Statistical analysis

We conducted a descriptive analysis using means, standard deviations, medians, and ranges for quantitative variables, and frequencies and percentages for qualitative variables. Categorical variables were analyzed in both groups using the Mantel Haenszel chi-square method or, where applicable, Fisher's exact test. The Kolmogorov–Smirnov method was used to test quantitative data for normality of distribution, and Student’s t-test and the Mann-Whitney U test were used to compare means for parametric and non-parametric data, respectively. Differences in LOS were analyzed by Kaplan-Meier survival curves and the curves for the two groups were compared with the Mantel-Cox log rank test. Two-tailed tests were used. The relative risks, with the 95% confidence interval, of prolonged hospital stay associated to each treatment (i.e. > 4 days), was calculated. The sample size calculation for the logrank test was generated by Lachin’s technique [28]. We assumed an exponential distribution, with an estimated hazard ratio of 0.126 in the placebo group versus 0.01 in the epinephrine group. We set the confidence interval at 95%, and calculated for a significance level of 95% (p<0.05) with a power of 80%, expecting no differences in sample size between groups. With these parameters, the sample size was estimated to be of 208 patients (104 per group).

## Results

**Fig 1:**
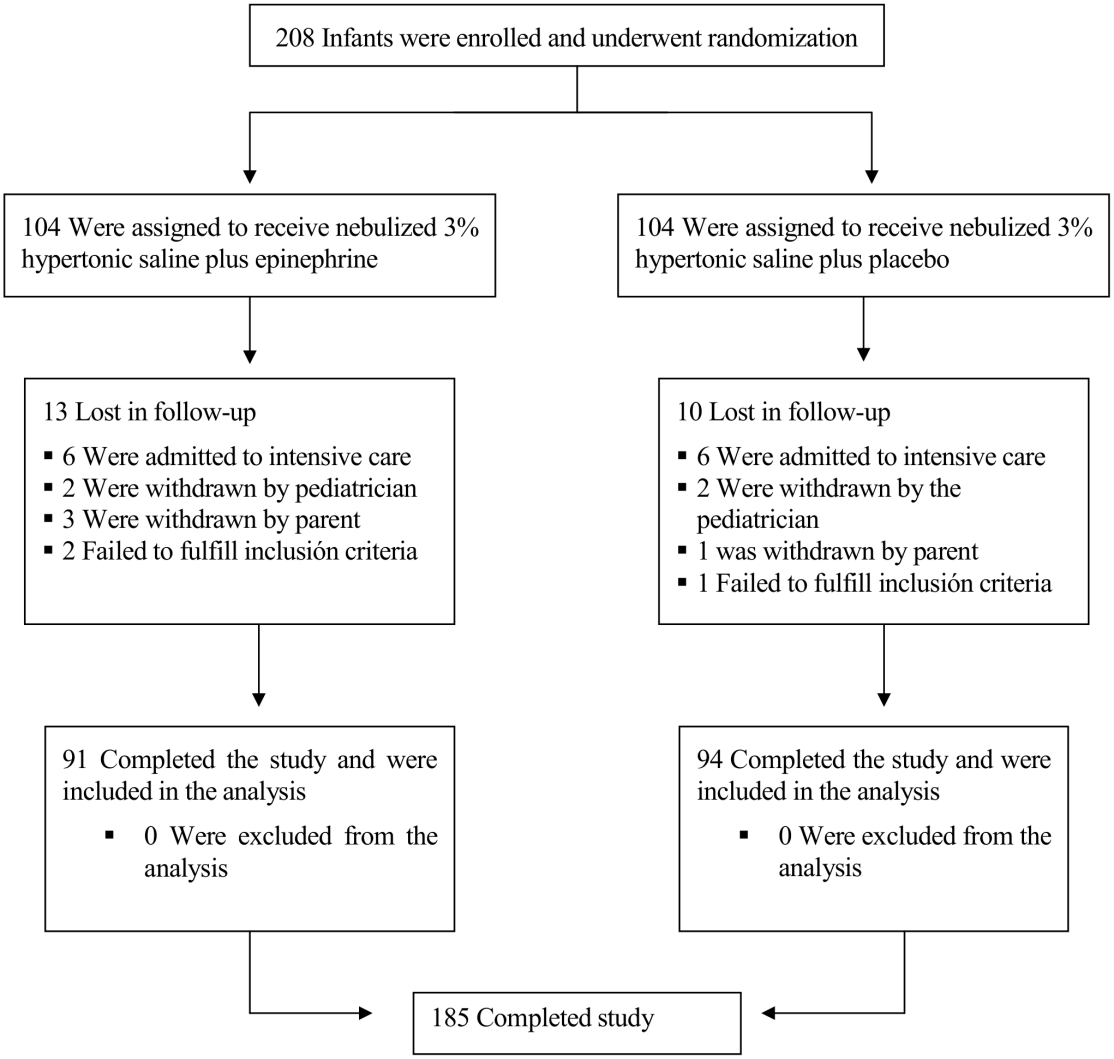
Randomization of the Study Patients.

A total of 208 infants with acute moderate bronchiolitis were enrolled in the study over a period of 3 epidemic years. Of these, 23 patients (10 in the hypertonic saline plus epinephrine group and 13 in the hypertonic saline plus placebo group) prematurely discontinued study treatment. The reasons for these dropouts were the use of non-study medications in 16 patients (8 in each group), withdrawal of consent by parents in four cases, and failure to fulfill inclusion criteria after enrollment in three cases (Fig 1). Consequently, 185 patients (94 in the epinephrine group and 91 in placebo group) were included in the analysis (S3 File: Flow diagram). Full data set provided as supporting information (S4 File: Full data set)

The mean age of the whole sample was 2.11 ± 2.22 months (95% CI 1.80–2.43) and 49.7% were male. There were 128 patients under 3 months (64 in each group), 45 patients aged 4–6 months (25 in epinephrine group and 20 in the placebo group), and 12 patients aged over 7 months (5 and 7, respectively).

No differences were found between the groups in any demographic characteristic, the presence of personal or family history of atopy, parental smoking, or the type of feeding. There were also no significant differences in WDF score on admission (i.e. enrollment), or in the rate of RSV positivity, or in the use of previous treatments (Table 2).

**Table 2:** Baseline Characteristics of Study Patients.

	3% Hypertonic Saline + Epinephrine	3% Hypertonic Saline + Placebo	P Value
N	94	91	
Mean age—months	2.10±2.37	2.12±2.08	0.678
Male sex—n°. [%]	46 (48.9)	46 (50.5)	0.826
Personal history of atopy—n°. [%]	19 (20.2)	15 (16.4)	0.240
Personal history of cow’s milk protein allergy—n°. [%]	1 (1)	1 (1)	0.621
Premature birth older than 6 weeks ^a^ —n°. [%]	6 (6.3)	11 (12)	0.171
Parental history of smoking—n°. [%]	25 (26.6)	32 (35.1)	0.226
Family history of atopy—n°. [%]	30 (31.9)	27 (29.6)	0.741
Breastfed—n°. [%]	50 (53.2)	51 (56)	0.697
Number of siblings	0.65±0.599	0.77±0.684	0.284
Attendance at a nursery school—n°. [%]	10 (10.6)	15 (16.5)	0.245
Disease severity score^b^ at admission	5.36±0.98	5.24±1.17	0.260
Respiratory syncytial virus positivity—n°. [%]	58 (61.7)	54 (59.3)	0.576
Previous treatment with salbutamol—n°. [%]	36 (38.3)	32 (35.1%)	0.659
Previous treatment with corticosteroids—n° [%]	27 (28.7)	22 (24.1)	0.483

Plus–minus values are means ±SD. N is number. No significant differences were found between the two groups in any of theses characteristics at enrollment.

^a^ Premature infant older than 6 weeks at the time of enrollment (not an exclusion criteria).

^b^ Defined by the Wood-Downes Scale modified by Ferres (WDF score).

### Primary efficacy outcome

**Fig 2:**
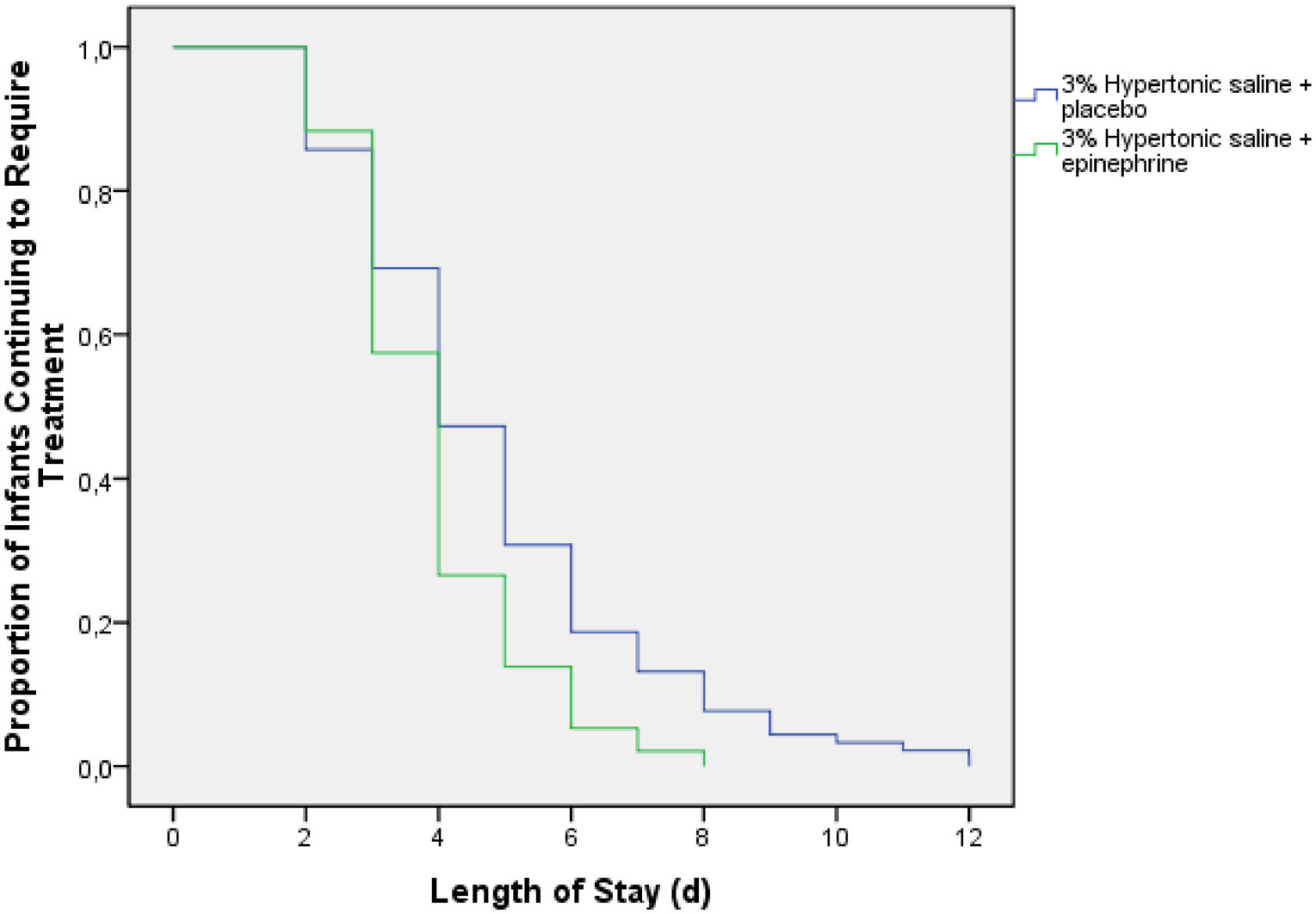
Length of hospital stay according to treatment. Kaplan–Meier plots show the proportion of infants who continued to require in-hospital treatment.

The mean hospital stay was shorter in the hypertonic saline plus epinephrine group (3.94±1.37) than in the hypertonic saline plus placebo group (4.82±2.3) (P = 0.011). The maximum duration of hospitalization was also shorter in the group that received epinephrine (8 days vs. 12 days). A significantly higher proportion of infants who received hypertonic saline plus placebo required hospitalization for more than 4 days (30.8% vs. 13.8% in the epinephrine group) (P = 0.006). The relative risk of a prolonged hospital stay in the epinephrine group was a half of the one in the placebo group (RR: 0.45, IC95%: 0.25–0.81). Comparison of survival curves showed significant differences in the LOS from day 4 onwards (P = 0.001) (Fig 2).

### Secondary efficacy outcomes

**Fig 3:**
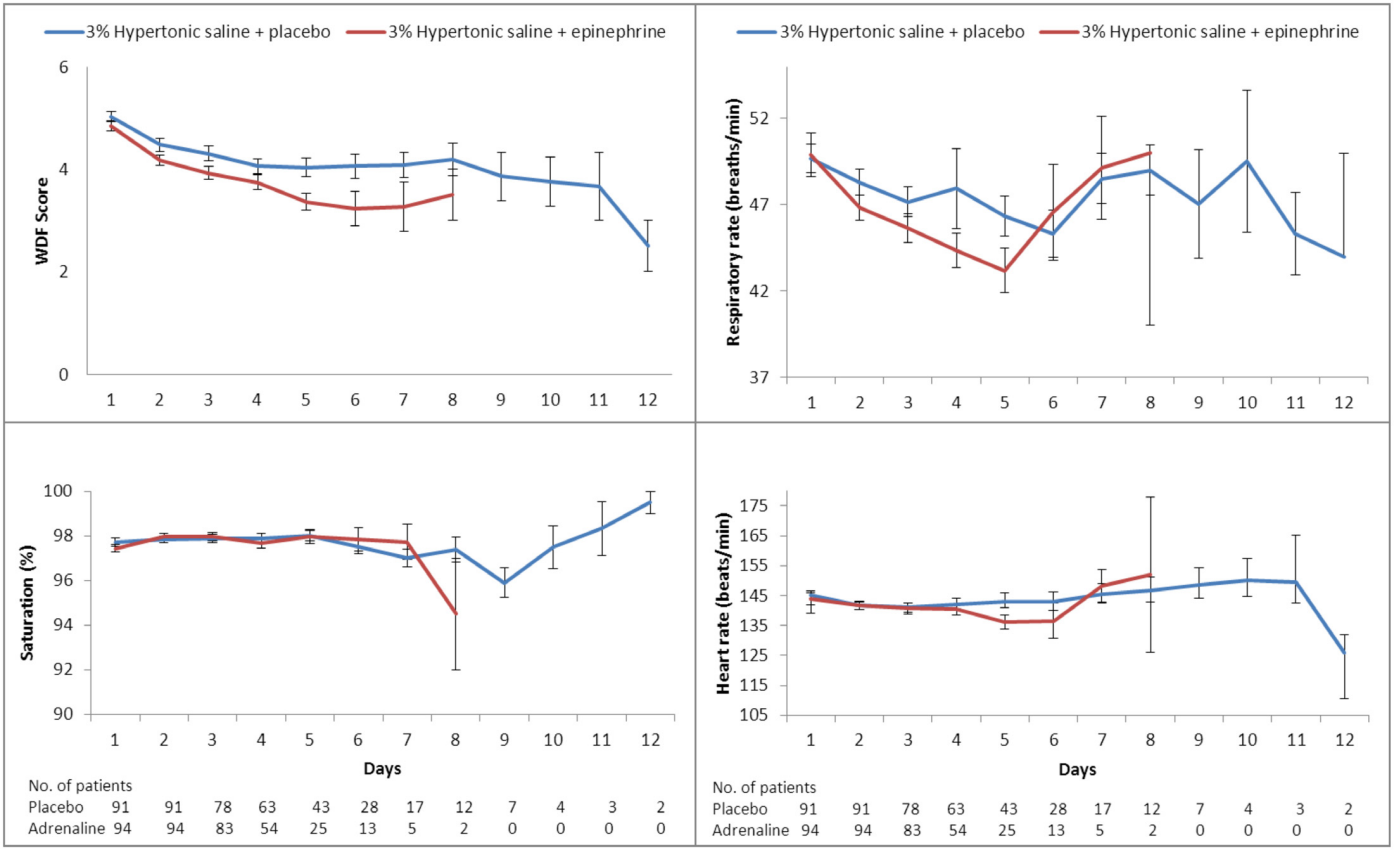
Mean secondary efficacy outcome values in both groups during hospitalization. WDF denotes Wood-Downes clinical score modified by Ferres.

Changes in secondary outcome variables (WDF score, respiratory rate, heart rate, oxygen saturation, and FiO2) are shown in Fig 3. The WDF score was similar in both groups on admission and improved more rapidly in the hypertonic saline plus epinephrine group, with significant differences between-groups observed already at day 3 (placebo group: mean 4.31, 95% CI (4.01–4.59); epinephrine group: mean 3.93, 95% CI (3.68–4.17), p = 0.029), which was sustained by day 5 (placebo group: mean 4.03, 95% CI (3.67–4.40); epinephrine group: mean 3.37, 95% CI (3.02–4.72), p = 0.036). Likewise, respiratory rate (as a marker of respiratory distress), was decreased in the epinephrine group vs. the placebo group during the first five days. Heart rate and oxygen saturation remained similar in both groups throughout the study (Fig 3). We found no adverse events (i.e. tachycardia, sweating, pallor, trembling, or hypertension), during hospitalization.

## Discussion

To the best of our knowledge, this is the first clinical trial specifically designed to evaluate the efficacy of added epinephrine to 3% hypertonic saline in hospitalized patients with acute moderate bronchiolitis. Our results show that the addition of epinephrine significantly shortens hospital stay in infants with acute bronchiolitis of moderate intensity (P = 0.011) in our setting. We also found that the relative risk of prolonged hospital stay in the group receiving epinephrine was half as much as in the group receiving placebo. Furthermore, infants who received epinephrine improved faster than those who did not.

In a preliminary report published by our group, analyzing data from a third of the final estimated sample, we already found a trend—though non significant—to an earlier clinical recovery in the epinephrine group by day three of hospitalization, together with improvement in clinical severity (p = 0.063) and respiratory rate (p = 0.096), without any adverse events. These preliminary data encouraged us to complete the study even though no significant differences in LOS were found yet [29].

When interpreting ours and others’ results in mean LOS for acute bronchiolitis, the typical duration of hospital stay where the study is conducted is a relevant factor to consider. There are settings in which the duration of hospital stay typically exceeds 3 days, and others in which it is typically shorter. Whether if this difference is due to the bronchiolitis etiology, or to populations’ vulnerability, or to any other factors such as the type of Health System (i.e. public versus private), has not been analyzed, but all of these may well influence. Our results show no difference during the first three days of treatment, which is in accordance with all the recent evidence of uselessness of any therapy tested in those settings where LOS is less than three days [12;30]. However, we found a clear and significant improvement in the group receiving epinephrine from the third day onwards, and the probability to need hospitalization for more that 4 days doubles when no epinephrine is added to the hypertonic nebulizations.

The combination of epinephrine and hypertonic saline was also associated with significantly improved WDF scores on days 3 and 5 of hospital treatment, reflecting the faster relief of respiratory distress in this group, and explaining the shorter time till discharge. A greater improvement was seen in respiratory rate over the first five days of hospitalization in infants who received epinephrine, but the difference with the group receiving placebo, did not reach significance. The results after day 5 are more difficult to interpret due to the smaller sample size in both groups. Heart rate was similar in both groups throughout the study, and we didn’t encounter any episode of post-nebulization tachycardia requiring discontinuation of treatment in infants receiving epinephrine.

In a very recent systematic review of the use of nebulized 3% hypertonic saline in infants with acute bronchiolitis, which analyzed 11 double-blind randomized clinical trials comparing nebulized 3% hypertonic saline versus normal saline in a total of 1090 patients, the hypertonic solution was found to be associated with significantly shorter LOS (P<0.001) and decreased disease severity, in the absence of relevant adverse effects [31], but these results must be interpreted cautiously in settings where the typical LOS is under 3 days, as it has been shown by others that hypertonic saline in such settings lacks efficacy [32;33]. Many other authors’ findings support the use of 3% hypertonic saline,[30;34;35] and conflicting reports are rare [36], but the latest review supports that nebulized 3% saline among inpatients (but not in the emergency department setting) decreased hospital LOS [37].

As mentioned above, it has also been studied if the use of epinephrine along with hypertonic at the Emergency Department would reduce the hospitalization rates, but this study found no beneficial effect [35], which is consistent with our results where the added value of epinephrine shows primarily after 3 days of treatment.

Most studies on 3% hypertonic saline have analyzed its efficacy in combination with certain bronchodilators. In one study, the treating physicians were free to add a bronchodilator to the hypertonic saline. In this study, most physicians chose to use a bronchodilator, thus the sample not receiving any bronchodilator was too limited to allow for comparison between groups receiving each bronchodilator versus those receiving only hypertonic saline [38]. A very recent report, showed a minimal reduction in hospital stay, in a group receiving albuterol in 3% saline when compared with albuterol in 0.9% saline (3.16 days versus 3.92), though the difference did not reach statistical significance [26]. The difference we found (3.94 vs 4.82 days), resulted significant, and—in our opinion—clinically relevant, and may be primarily explained by the higher proportion of infants needing prolonged hospitalization in the placebo group.

Our results may not be extrapolated to settings in which the average LOS is typically shorter than 3 days, but may well be taken into account in places where the duration of hospital stay normally exceeds this time.[22,23,24]

## Conclusions

Nebulized epinephrine in 3% saline significantly shortens the length of hospital stay of infants with acute moderate bronchiolitis in our setting, where it normally exceeds 4 days, and reduces the risk of a prolonged stay, without any increase in the occurrence of adverse events, when compared with placebo in 3% saline.

## Supporting Information

S1 File CONSORT 2010 Compliance Checklist. (PDF)

S2 File Study Protocol (FULL ORIGINAL VERSION). (PDF)

S3 File Flow diagram CONSORT 2010. (PDF)

S4 File Full data set (SPSS version). (SAV)
