# Supplementary material for: Epinephrine Improves the Efficacy of Nebulized Hypertonic Saline in Moderate Bronchiolitis: A Randomised Clinical Trial
Source: PLoS One. 2015 Nov 17;10(11):e0142847. doi: 10.1371/journal.pone.0142847 (PMC4648584; doi:10.1371/journal.pone.0142847)
Supplement: S2 File — (PDF) [file pone.0142847.s002.pdf]

JCFG-2010-SSH3%

ENSAYO CLÍNICO ALEATORIZADO, DOBLE CIEGO, CONTROLADO Y PARALELO PARA COMPARAR LA EFICACIA DE LA NEBULIZACIÓN DE SUERO SALINO HIPERTÓNICO AL 3% CON ADRENALINA FRENTE A NEBULIZACIÓN DE SUERO SALINO HIPERTÓNICO AL 3% EN NIÑOS HOSPITALIZADOS CON BRONQUIOLITIS AGUDA MODERADA

Código del protocolo: JCFG-2010-SSH3%

Nº EudraCT: 2009-016042-57

Versión: v 1.2

Fecha: 26-01-2012

Promotor: Jose Carlos Flores González

Página de firmas de aprobación del protocolo

Título del protocolo: Ensayo clínico aleatorizado, doble ciego, controlado y paralelo para comparar la eficacia de la nebulización de suero salino hipertónico al 3% con adrenalina frente a nebulización de suero salino hipertónico al 3% en niños hospitalizados con bronquiolitis aguda moderada

N.º de protocolo: JCFG-2010-SSH3%

Aceptación del protocolo por el investigador

Facilitaré copias del protocolo del estudio y acceso a toda la información relacionada con el mismo al personal del estudio de mi centro que guarde relación con este protocolo. Les explicaré este material para garantizar que están plenamente informados acerca de los medicamentos en investigación y que entienden el protocolo.

Todo el equipo investigador ha leído detenidamente y entiende este protocolo y acepta realizar este estudio con arreglo al protocolo adjunto, a la Buena práctica clínica (BPC), la Conferencia Internacional de Armonización (ICH) y todas las leyes y normativa nacional y locales vigentes, así como con los requisitos del comité ético de investigación clínica (CEIC) pertinente y cualquier otro requisito institucional. No se efectuarán modificaciones del protocolo del estudio sin la autorización previa del CEIC.

He leído, entiendo y me comprometo a acatar todas las condiciones e instrucciones contenidas en este protocolo.

Firma del investigador principal

José Carlos Flores González

Firma investigador colaborador

Teresa Domínguez Coronel

Firma investigador colaborador

Miriam Aragón Ramírez

Firma investigador colaborador

Miguel Angel Matamala Morillo

Firma investigador colaborador

Rosa Mª García Ortega

Firma investigador colaborador

Patricia Rodríguez Campoy

Firma investigador colaborador
Jesus Carrera Martínez

Firma investigador colaborador
Rocío Bulo Concellón

Firma investigador colaborador
Mª Victoria García Palacios

Fecha

RESUMEN

1.1 SOLICITUD

Solicitud de un ensayo clínico sin calificación de PEI

1.2 PROMOTOR

José Carlos Flores González

1.3 TÍTULO DEL ENSAYO

ENSAYO CLÍNICO ALEATORIZADO, DOBLE CIEGO, CONTROLADO Y PARALELO PARA COMPARAR LA EFICACIA DE LA NEBULIZACIÓN DE SUERO SALINO HIPERTÓNICO AL 3% CON ADRENALINA FRENTE A NEBULIZACIÓN DE SUERO SALINO HIPERTÓNICO AL 3% EN NIÑOS HOSPITALIZADOS CON BRONQUIOLITIS AGUDA MODERADA

1.4 CÓDIGO DEL PROTOCOLO

JCFG-2010-SSH3%

1.5 Nº EUDRACT

2009-016042-57

1.6 VERSIÓN Y FECHA

V 2.1 del 16 de abril de 2012

1.7 INVESTIGADOR COORDINADOR

Jose Carlos Flores González

1.8 CENTROS DONDE SE PREVÉ REALIZAR EL ESTUDIO

Hospital Universitario Puerta del Mar (Cádiz)

1.9 COMITÉ ÉTICO DE INVESTIGACIÓN CLÍNICA QUE LO EVALÚA

El estudio se presentará al Comité Autonómico de Ensayos Clínicos y al Comité Local de Ensayos Clínicos del Hospital Universitario Puerta del Mar.

1.10 Monitor del estudio

Por identificar. Se contratará a una CRO para que desarrolle las labores de monitorización del estudio.

1.11 Tratamiento en estudio

Tratamiento experimental: Suero salino hipertónico al 3% en nebulización

Tratamiento de control: suero salino hipertónico al 3% + adrenalina en nebulización

Descripción del tratamiento:

La medicación nebulizada para el grupo control consistirá en 3 ml de adrenalina 1:1000 más 5.5 ml de agua destilada más 1.5 ml de suero hipertónico al 20%, con un total de 10 ml.

La medicación nebulizada para el grupo experimental consistirá en 8.5 ml de agua destilada más 1.5 ml de suero hipertónico al 20% con un total de 10 ml.

Ambas soluciones de nebulización tiene una concentración final de sodio del 3%. De inicio todos los pacientes recibirán nebulizaciones cada 4 horas. Según la evolución clínica, las nebulizaciones se podrán administrar hasta un máximo de cada 3 horas si la evolución no es satisfactoria o bien se irán espaciando a cada 6 y posteriormente a cada 8 horas hasta el alta médica si la evolución es satisfactoria.

1.12 Objetivo principal

El objetivo principal de este estudio es analizar si la estancia hospitalaria de los pacientes tratados con nebulizaciones de suero salino hipertónico al 3% es igual o menor que la de los pacientes tratados con nebulizaciones de suero salino hipertónico al 3% más adrenalina.

1.13 Diseño

Ensayo clínico unicéntrico, prospectivo, aleatorizado, doble ciego, controlado y paralelo.

1.14 Enfermedad o trastorno en estudio

Bronquiolitis aguda moderada

1.15 VARIABLE PRINCIPAL DE VALORACIÓN

Estancia hospitalaria, definida como el tiempo que transcurre desde que el paciente cumple los criterios de inclusión en el estudio hasta que cumple los criterios de finalización.

1.16 POBLACIÓN EN ESTUDIO Y NÚMERO TOTAL DE SUJETOS

Serán incluidos en el estudio de forma prospectiva, todos los lactantes menores de 24 meses ingresados con el diagnóstico de bronquiolitis aguda moderada (Clasificación Internacional de Enfermedades (CIE), novena revisión. Código 466.1) en los próximos 3 años epidémicos.

Número total de sujetos: 200 pacientes entre ambos grupos en proporción 1:1

1.17 CALENDARIO

Inicio del estudio: Octubre del 2010, incluido

Desarrollo del estudio: 3 años epidémicos consecutivos (2010-2011, 2011-2012 y 2012-2013)

Finalización del estudio: Abril 2013, incluido

ÍNDICE

1 Resumen ____ *4*

1.1 Solicitud ____ 4

1.2 Promotor ____ 4

1.3 Título del ensayo ____ 4

1.4 Código del protocolo ____ 4

1.5 Nº EudraCT ____ 4

1.6 Versión y fecha ____ 4

1.7 Investigador coordinador ____ 4

1.8 Centros donde se prevé realizar el estudio ____ 4

1.9 Comité ético de investigación clínica que lo evalúa ____ 4

1.10 Monitor del estudio ____ 5

1.11 Tratamiento en estudio ____ 5

1.12 Objetivo principal ____ 5

1.13 Diseño ____ 5

1.14 Enfermedad o trastorno en estudio ____ 5

1.15 Variable principal de valoración ____ 6

1.16 Población en estudio y número total de sujetos ____ 6

1.17 Calendario ____ 6

2 Información General ____ *10*

2.1 Título del protocolo ____ 10

2.2 Código de Protocolo ____ 10

2.3 Nº EudraCT ____ 10

2.4 Versión ____ 10

2.5 Datos del Promotor y Monitor ____ 10

2.6 Identificación de los expertos médicos del promotor del ensayo ____ ¡Error! Marcador no definido.

2.7 Identificación de los investigadores y centros ____ ¡Error! Marcador no definido.

3 Justificación ____ *11*

3.1 BRONQUIOLITIS ____ 11

3.2 Fármacos del estudio ____ 11

3.3 Justificación del estudio ____ 11

3.4 Descripción de la vía de administración, dosis, pauta de ____ 12

3.4 dosificación y periodo de tratamiento ____ 12

3.5 Declaración de que el ensayo será realizado de acuerdo con el protocolo, las buenas prácticas clínicas y los requisitos legales pertinentes ____ 13

3.6 Descripción de la población de estudio ____ 14

4 Objetivos del Ensayo _______________ *16*

4.1 Objetivo principal _______________ **16**

4.2 Objetivos secundarios _______________ **16**

4.3 Objetivo exploratorio SI PORCEDE _______________ ¡Error! Marcador no definido.

5 Diseño del Ensayo _______________ *16*

5.1 Descripción específica de las variables principales y secundarias que se evaluarán en el ensayo _______________ **16**

5.1.1 Variable principal _______________ **¡Error! Marcador no definido.**

5.1.2 Variables secundarias _______________ **¡Error! Marcador no definido.**

5.1.3 Evaluación de la seguridad _______________ 17

5.2 Descripción del tipo/diseño del ensayo _______________ **18**

5.3 Técnicas de enmascaramiento _______________ **18**

5.4 Periodos del estudio _______________ **18**

5.4.1 Fases del estudio _______________ 18

5.4.2 Desarrollo del estudio: Visitas y procedimientos _______________ 19

5.5 Criterios de interrupción o finalización del ensayo _______________ **25**

5.6 Procedimientos para contabilizilar el medicamento en investigación _______________ **25**

5.7 Identificación de datos recogidos directamente en el cuaderno de recogida de datos ¡Error! Marcador no definido.

6 Selección y retirada de sujetos _______________ *25*

6.1 Criterios de inclusión de los sujetos _______________ **25**

6.2 Criterios de exclusión de los sujetos _______________ **25**

6.3 Criterios de retirada de los sujetos _______________ **26**

7 Tratamiento de los sujetos _______________ *26*

7.1 Tratamiento de estudio _______________ **26**

7.1.1 Descripción del tratamiento del estudio _______________ 26

7.1.2 Duración del tratamiento _______________ 26

7.1.3 Instrucciones de uso y administración del tratamiento _______________ 26

7.1.4 Modificación de los esquemas del tratamiento por acontecimientos adversos _______________ 27

7.1.5 Procedimientos para monitorizar el cumplimiento del sujeto _______________ 27

7.1.6 Destrucción de la medicación del estudio _______________ 27

7.2 Medicamentos o tratamientos permitidos y no permitidos antes de y/o durante el ensayo _______________ **28**

7.2.1 Tratamientos permitidos _______________ 28

7.2.2 Tratamientos no permitidos _______________ 28

7.2.3 Medicación de rescate _______________ 28

8 Valoración de la eficacia _______________ *28*

8.1 Especificación de los parámetros de eficacia _______________ **28**

8.1.1 Parámetro principal de eficacia _______________ 28

8.1.2 Parámetros secundarios de eficacia _______________ 28

8.2 Los métodos y el calendario para la evaluación, registro y análisis de los parámetros de eficacia _______________ **29**

9 Valoración de seguridad _______________ *29*

9.1 Especificación de los parámetros de seguridad _______________ **29**

9.2 Métodos y el calendario para la evaluación, registro y análisis de los parámetros de seguridad 29
9.2.1 Acontecimiento adverso, definición 30
9.2.2 Recogida y notificación de acontecimientos adversos 30
9.3 Seguimiento de los sujetos después de los acontecimientos adversos 32
10 Estadística 33
10.1 Descripción de los métodos estadísticos que se usarán, incluyendo el calendario de todos los análisis intermedios planificados 33
10.2 Número previsto de sujetos que se incluirán 33
10.3 Nivel de significación que se utilizará 33
10.4 Criterios para la finalización del ensayo 34
10.5 Procedimiento utilizado para contabilizar los datos perdidos, no utilizados y erróneos 34
10.6 Selección de los sujetos que se van a incluir en cada análisis ¡Error! Marcador no definido.
11 Acceso Datos/Documentos Fuente 35
12 Control y Garantía de Calidad 35
12.1 Monitorización del estudio 35
12.2 Auditorias e inspecciones 35
13 Ética 35
13.1 Legislación / Declaración de Helsinki / ICH 35
13.2 Información a los participantes del estudio 36
14 Manejo de los Datos y Archivo de los Registros 36
14.1 Confidencialidad 36
14.2 Uso de medios electrónicos ¡Error! Marcador no definido.
15 Financiación y Seguros 36
16 Política de Publicación 36
ANEXO 1. Calendario de evaluaciones 38
ANEXO 2. Modelo consentimiento informado 42

Información General

1.18 Título del protocolo

ENSAYO CLÍNICO ALEATORIZADO, DOBLE CIEGO, CONTROLADO Y PARALELO PARA COMPARAR LA EFICACIA DE LA NEBULIZACIÓN DE SUERO SALINO HIPERTÓNICO AL 3% CON ADRENALINA FRENTE A NEBULIZACIÓN DE SUERO SALINO HIPERTÓNICO AL 3% EN NIÑOS HOSPITALIZADOS CON BRONQUIOLITIS AGUDA MODERADA

1.19 Código de Protocolo

JCFG-2010-SSH3%

1.20 Nº EudraCT

2009-016042-57

1.21 Versión

V 2.1 del 16 de abril de 2012

1.22 Datos del Promotor y Monitor

Promotor: J Carlos Flores González

Investigadores principal: J Carlos Flores González

Investigadores colaboradores: Miriam Aragón Ramírez, Teresa Domínguez Coronel, Arturo Hernández González, Rocío Bulo Concellón y Tomás Aguirre Copano

Monitorización: El promotor del estudio contratará una CRO que será la responsable de monitorizar el estudio.

JUSTIFICACIÓN

1.23 BRONQUIOLITIS

La bronquiolitis se define clínicamente como el primer episodio de sibilancias y dificultad respiratoria precedido de un cuadro de infección de vías respiratorias altas (rinorrea, estornudos y tos con o sin fiebre no muy alta) durante la época epidémica (generalmente de noviembre a marzo) en un lactante menor de 2 años.

La bronquiolitis es la causa más frecuente de infección respiratoria de vías bajas en los lactantes (1) y la causa más frecuente de ingreso hospitalario en menores de 1 año. El virus respiratorio sincitial (VRS) es el principal agente etiológico, aislándose hasta en el 80 % de las ocasiones. Según el Centro de Control y Prevención de Enfermedades (CDC), entre un 25 y un 40% de los lactantes y niños pequeños que se exponen por primera vez al virus respiratorio sincitial (VRS) manifiestan signos y síntomas de bronquiolitis o neumonía. Además, la infección por VRS no produce inmunidad permanente, pudiéndose producir reinfecciones durante toda la vida, aunque estas suelen ser más banales. Hall et al, describen una tasa de reinfección de hasta el 73% en los siguientes 26 meses tras una primera infección. Casi todos los niños habrán padecido al menos una infección por el VRS a la edad de 2 años.

1.24 FÁRMACOS DEL ESTUDIO

Adrenalina nebulizada

Suero salino hipertónico al 3 % nebulizado

1.25 JUSTIFICACIÓN DEL ESTUDIO

A pesar de la frecuencia e importancia de esta infección respiratoria, aún existen grandes controversias sobre el protocolo terapéutico más adecuado; muchos estudios reflejan una amplia variación en el diagnóstico y tratamiento de la bronquiolitis que corresponden más a preferencias hospitalarias o individuales que a la gravedad del episodio (5). También hay que considerar que el hecho de que la terapéutica sea sólo de soporte y no curativa unido a que la mayoría de los niños con bronquiolitis evoluciona bien independientemente del tratamiento elegido, conlleva a que se persista en la utilización de medicamentos que aportan poco o ningún beneficio (5). Pero no debemos de olvidar que esta falta de consenso en la práctica clínica repercute tanto en los costes como en las estancias de hospitalización (6)

Según los estudios publicados hasta la fecha, la adrenalina nebulizada parece ser discretamente más beneficiosa que otros broncodilatadores nebulizados, aún así su mejoría en las escalas clínicas son controvertidas, y ningún estudio concluye que reduzca la estancia hospitalaria (7-11), por lo que las guías de práctica clínica no recomiendan su uso de forma rutinaria (1). Los efectos adversos de la nebulización de adrenalina más destacables son el

aumento de forma significativa de la frecuencia cardiaca y de forma leve y no significativa la frecuencia respiratoria y la tensión arterial (8). Estos efectos adversos generalmente no son lo suficientemente importantes como para interrumpir el tratamiento.

En los últimos años se han publicado varios ensayos clínicos aleatorizados y revisiones sistemáticas comparando la nebulización de broncodilatadores diluidos en suero fisiológico con las nebulizaciones de broncodilatadores diluidos en suero hipertónico al 3%, demostrando una reducción en la estancia hospitalaria (12-16).

El suero salino hipertónico ejerce su efecto a través de varios mecanismos: a) Rompe los enlaces iónicos dentro del gel mucinoso, reduciendo el grado de enlace cruzado y la densidad de su trama (17) disminuyendo así la viscosidad y elasticidad de la secreción mucosa. b) Provoca un flujo osmótico de agua hacia el interior de la capa de moco, rehidratando así las secreciones y mejorando la reología del moco (18). c) Estimula la movilidad ciliar mediante la liberación de prostaglandina E2 (19). d) Puede causar también la inducción de tos y esputo, lo que puede ayudar a aclarar vía aérea (12).

Pero el suero salino hipertónico no está exento de efectos adversos, de forma que a alta concentración (mayor o igual al 4.5%) puede causar broncoconstricción en pacientes asmáticos y por tanto se ha recomendado su administración junto a un broncodilatador (20). Otros estudios sin embargo, muestran que la nebulización de suero salino hipertónico al 4,5 e incluso al 7% sin broncodilatador es totalmente segura tanto en niños no asmáticos como en niños con obstrucción de las vías aéreas pequeñas secundaria a la fibrosis quística (21, 22).

En nuestra revisión bibliográfica no hemos encontrado hasta la fecha, ningún artículo que compare la eficacia y seguridad de la administración de nebulizaciones de broncodilatadores diluidos en suero salino hipertónico al 3% de la administración de nebulizaciones de suero salino hipertónico al 3% en los pacientes hospitalizados con el diagnóstico de bronquiolitis. En el único artículo que incluye un subgrupo que recibe nebulizaciones de suero salino hipertónico al 3% sin broncodilatador en este tipo de pacientes, no observan ningún efecto secundario aunque el tamaño de la muestra es pequeño (13).

1.26 Descripción de la vía de administración, dosis, pauta de dosificación y periodo de tratamiento

Tratamiento experimental: Suero salino hipertónico al 3% en nebulización

Tratamiento de control: suero salino hipertónico al 3% + adrenalina en nebulización

Descripción del tratamiento: Un grupo control recibirá nebulizaciones de adrenalina más suero salino hipertónico al 3% y un grupo de estudio recibirá nebulizaciones de placebo más suero salino hipertónico al 3%. La medicación nebulizada consistirá en 3 ml de adrenalina 1:1000 más 5.5 ml de agua destilada

más 1.5 ml de suero hipertónico al 20%, con un total de 10 ml, para el grupo control; y de 8.5 ml de agua destilada más 1.5 ml de suero hipertónico al 20% con un total de 10 ml, para el grupo de estudio. Ambas soluciones de nebulización tiene una concentración final de sodio del 3%. De inicio todos los pacientes recibirán nebulizaciones cada 4 horas. Según la evolución clínica, las nebulizaciones se podrán administrar hasta un máximo de cada 3 horas si la evolución no es satisfactoria o bien se irán espaciando a cada 6 y posteriormente a cada 8 horas hasta el alta médica si es satisfactoria

1.27 DECLARACIÓN DE QUE EL ENSAYO SERÁ REALIZADO DE ACUERDO CON EL PROTOCOLO, LAS BUENAS PRÁCTICAS CLÍNICAS Y LOS REQUISITOS LEGALES PERTINENTES

El presente estudio clínico se realizará de acuerdo con el protocolo, los principios establecidos en la versión revisada actual de la Declaración de Helsinki (Seúl, Octubre 2008) y de acuerdo con los requisitos reguladores aplicables, en particular las Normas armonizadas tripartitas ICH para las Buenas Prácticas Clínicas 1996 y el Real Decreto de Ensayos Clínicos 223/2004, por el que se regulan los ensayos clínicos con medicamentos en España, y que incorpora en su totalidad el ordenamiento de la Directiva Europea 2001/20/CE, relativa a las disposiciones de los estados miembros sobre la aplicación de buenas prácticas clínicas en la realización de ensayos clínicos de medicamentos de uso humano.

El investigador consiente, cuando firma el protocolo, en adherirse a las instrucciones y procedimientos descritos en ellos y de esta manera, seguir los principios de las buenas prácticas clínicas que ellos implican.

En cumplimiento con el Real Decreto 223/2004, el promotor presentará la documentación pertinente al CEIC que escoja de referencia para su evolución y posterior informe. El estudio no podrá iniciarse hasta tener la aprobación escrita del CEIC de referencia, así como la autorización de la AEMPS.

Cualquier enmienda que cambie la relación beneficio riesgo para el paciente deberá ser, una vez firmada por el promotor, sometida a los CEICs y a la AEMPS para su aprobación. Los investigadores del estudio también serán informados y darán su aprobación escrita de la misma.

El consentimiento informado de cada sujeto será otorgado libremente antes de la participación en el ensayo clínico, bien por escrito o bien de forma oral ante testigos.

El personal del estudio involucrado en la realización de este ensayo estará suficientemente cualificado por su educación, formación y experiencia para llevar a cabo las tareas asignadas.

1.28 DESCRIPCIÓN DE LA POBLACIÓN DE ESTUDIO

Serán incluidos en el estudio de forma prospectiva todos los lactantes menores de 24 meses ingresados en la Unidad de Gestión Clínica de Pediatría del Hospital Universitario Puerta del Mar de Cádiz con el diagnóstico de bronquiolitis aguda moderada (Clasificación Internacional de Enfermedades (CIE), novena revisión. Código 466.1), durante los 3 próximos años epidémicos, desde el 1 de octubre de 2010 al 1 de mayo de 2013. Se realizarán análisis estadísticos de los resultados cada 2 meses, de manera acumulativa, para detectar lo más precozmente posibles diferencias significativas entre los dos grupos de tratamiento y decidir sobre la continuidad del estudio.

Los criterios de inclusión son:

1.- Lactante menor de 24 meses sin patología de base y con necesidad de ingreso hospitalario por el diagnóstico clínico de bronquiolitis aguda, definida como el primer episodio de sibilancias asociada a un cuadro de infección de vías respiratorias altas durante la época epidémica.

2.- La bronquiolitis debe ser de gravedad moderada, definida como aquellos pacientes que presenten una frecuencia respiratoria mayor de 60 y menor de 70, una puntuación en la escala de Wood-Downes modificada entre 4 y 7, ambos incluidos, acidosis respiratoria con retención de carbónico ($pO_2 < 60$ mmHg, $pCO_2 > 45$ mmHg), hipoxemia ($SatO_2 < 92\%$ con FiO_2 ambiental) o rechazo de tomas.

3.- Consentimiento informado libremente otorgado por los padres o tutores.

Los criterios de exclusión serán:

1.- Enfermedad respiratoria crónica como anomalías congénitas, displasia broncopulmonar o fibrosis quística.

2.- Cardiopatía hemodinámicamente significativa

3.- Inmunodeficiencia

4.- Episodio anterior de sibilancias o diagnostico previo de asma

5.- Antecedentes de prematuridad menor de 37 semanas de gestación con edad corregida de menos de 6 semanas

6.- Haber recibido tratamiento durante el ingreso con corticoides sistémicos o inhalados

7.- Enfermedad neuromuscular

8.- Pacientes con ventilación mecánica domiciliaria

9.- Pacientes con bronquiolitis grave que requieran ingreso en UCIP.

10.- Cualquier otra patología grave que a juicio del investigador contraindique la participación en el estudio

11.- Participación en estudios de investigación en los seis meses anteriores al momento del reclutamiento

El protocolo de manejo diagnóstico-terapéutico de la bronquiolitis está escrito, consensuado y a disposición de todos los facultativos especialistas en pediatría, residentes de pediatría y del personal de enfermería responsable de sus cuidados.

OBJETIVOS DEL ENSAYO

1.29 OBJETIVO PRINCIPAL

El objetivo principal de este estudio es analizar si la estancia hospitalaria de los pacientes tratados con nebulizaciones de suero salino hipertónico al 3% es igual o menor que la de los pacientes tratados con nebulizaciones de suero salino hipertónico al 3% más adrenalina.

1.30 OBJETIVOS SECUNDARIOS

Los objetivos secundarios son comparar si la evolución de la escala de gravedad, las constantes vitales (frecuencia cardiaca, frecuencia respiratoria y saturación de oxígeno), la evolución clínica y analítica (gasometría), los efectos adversos y la necesidad de ventilación mecánica de estos pacientes durante el ingreso son más favorables en un grupo que en otro.

DISEÑO DEL ENSAYO

1.31 DESCRIPCIÓN ESPECÍFICA DE LAS VARIABLES PRINCIPALES Y SECUNDARIAS

Variable principal:

Estancia hospitalaria: tiempo que transcurre desde que el pediatra que atiende al paciente realiza el diagnóstico de bronquiolitis aguda moderada, ya sea en el Sección de Urgencias de Pediatría o en la Planta de Hospitalización de la Unidad de Gestión Clínica de Pediatría, hasta que el paciente cumple los criterios de finalización del estudio definidos en el protocolo.

Variables secundarias:

Evolución según una escala de gravedad de Wood-Downes modificada
Constantes vitales: frecuencia cardiaca, respiratoria y saturación de oxígeno
Evolución clínica en el estado general, apetito y auscultación
Evolución de los parámetros gasométricos
Efectos adversos: taquicardia, sudoración, palidez, temblor, hipertensión, tos, dificultad respiratoria, taquipnea.
Necesidad de ventilación mecánica

Definiciones

Bronquiolitis es el primer episodio de sibilancias y dificultad respiratoria precedido de un cuadro de infección de vías respiratorias altas (rinorrea, estornudos y tos con o sin fiebre no muy alta) durante la época epidémica (generalmente de noviembre a marzo) en un lactante menor de 2 años.

Para la valoración de la gravedad del episodio de bronquiolitis se empleará la escala de Wood-Downes modificado, que está compuesta por 6 items

(sibilancias, tiraje, frecuencia respiratoria, frecuencia cardiaca, entrada de aire y cianosis) que se puntúan de 0 a 3, siendo 0 la normalidad y 3 la gravedad. Según la puntuación obtenida se clasifica la bronquiolitis en leve (1-3 puntos), moderada (4-7 puntos) o severa (8-14 puntos).

Se define enfermedad pulmonar crónica o displasia broncopulmonar como aquel lactante menor de 32 semanas de gestación que ha precisado oxígeno suplementario durante 28 días o más evaluado a las 36 semanas de edad postmenstrual o al alta domiciliaria, o aquel lactante de 32 o más semanas de gestación que ha precisado oxígeno suplementario durante 28 días o más evaluado con más de 28 días pero menos de 56 días de vida o al alta a domicilio (1).

Cardiopatía hemodinámicamente significativa es aquella que recibe medicación para controlar la insuficiencia cardiaca, tiene hipertensión pulmonar moderada o severa o presenta una cardiopatía congénita cianosante.

La oxigenoterapia se administrará humidificada y con una FiO2 suficiente para mantener saturaciones de oxígeno por encima del 95%.

El aumento de la frecuencia cardiaca o taquicardia se valorará según las variaciones fisiológicas propias de la edad (< 6 m: > 180 lpm, 6-12 m: > 170 lpm, 1-2 años: > 150 lpm, 3-7 años: > 135 lpm, 8-11 años: > 130 lpm y 12-14 años: > 120 lpm)

La administración de antibióticos se reservará a aquellos niños con bronquiolitis que cumplan criterios de presentar una sobreinfección bacteriana.

Bronquiolitis moderada: Se considera bronquiolitis moderada aquellos que presenten una frecuencia respiratoria mayor de 60 y menor de 70, una puntuación en la escala de Wood-Downes modificada entre 4 y 7, ambos incluidos, acidosis respiratoria con retención de carbónico (pO2 < 60 mmHg, pCO2 > 45 mmHg), hipoxemia (SatO2 < 92% con FiO2 ambiental) o rechazo de tomas.

Criterios de finalización del estudio: tener una escala de gravedad ≤ 3 puntos, saturación de oxígeno > 97% respirando aire ambiente, adecuada tolerancia oral y la no necesidad de nebulizaciones. Si su estado empeora y se clasifica como bronquiolitis aguda grave, se retirará del estudio

Bronquiolitis grave es aquella que presenta una de las siguientes: una puntuación en la escala de Wood-Downes modificada mayor de 7, una saturación de oxígeno menor del 91% con una FiO2 mayor del 0.4, alteración del nivel de conciencia, episodios de apneas, empeoramiento rápido o alteraciones gasométricas importantes (pH menor de 7,10 o pCO2 mayor de 60) a pesar de un tratamiento óptimo.

1.31.1 Evaluación de la seguridad

Las evaluaciones de la seguridad incluirán la descripción de:

Acontecimientos adversos (AA), incluidos los AA de especial interés, como la taquicardia, bronconstricción, sudoración, temblor, palidez, taquipnea y hipertensión arterial

Constantes vitales: presión arterial, frecuencia cardíaca, frecuencia respiratoria y temperatura corporal
Datos de la exploración física
Parámetros analíticos habituales: bioquímica, hematología

Se prestará especial atención a los acontecimientos adversos graves y a los acontecimientos adversos inesperados

1.32 DESCRIPCIÓN DEL TIPO/DISEÑO DEL ENSAYO

Ensayo clínico aleatorizado, fase II, doble ciego, controlado y de dos grupos paralelos para comparar la eficacia y la seguridad de la nebulización de suero salino hipertónico al 3% con adrenalina frente a nebulización de suero salino hipertónico al 3% en niños hospitalizados con bronquiolitis aguda moderada

1.33 TÉCNICAS DE ENMASCARAMIENTO

La Unidad de Gestión Clínica de Farmacia, de acuerdo con el Art. 3.2 de la ORDEN SCO 256/2007, se encargará de preparar, envasar y etiquetar los medicamentos en investigación según la secuencia de aleatorización generada por ordenador identificándola con un código desconocido para médico y paciente. La medicación de ambos grupos tendrá el mismo volumen, peso, y características organolépticas

1.34 PERIODOS DEL ESTUDIO

1.34.1 FASES DEL ESTUDIO

Fase de inclusión de pacientes: Desde octubre de 2010 hasta abril de 2013, ambos inclusive, se incluirán todos los pacientes que ingresen en la Unidad de Gestión Clínica de Pediatría del Hospital Universitario Puerta del Mar de Cádiz con el diagnóstico de bronquiolitis aguda moderada, que cumplan los criterios de inclusión en el estudio y que sus padres o tutores consientan libremente en participar en el estudio.

Fase de tratamiento de los pacientes: A todo lactante que ingrese en observación o planta de hospitalización de pediatría y cumpla los criterios de ingreso, incluido la aceptación del consentimiento informado, se les asignará aleatoriamente un grupo (experimental o control) y se iniciará tratamiento con medicación nebulizada y unas medidas generales de soporte común para ambos grupos. Las nebulizaciones se administraran cada 4 horas, con la posibilidad de espaciarlas o aproximarlas según evolución clínica del paciente. Los pacientes finalizaran la fase de tratamiento si cumplen los criterios de finalización del estudio, si los padres o tutores legales deciden retirarlo del estudio o si el pediatra responsable decide su retirada.

1.34.2 Desarrollo del estudio: Visitas y procedimientos

1.34.2.1 Visita de Pre-Selección

Lactante menor de 2 años que acude a Urgencias de Pediatría del Hospital Universitario Puerta del Mar de Cádiz, por un proceso respiratorio de vías bajas durante los meses de octubre a abril de los años 2010-2011, 2011-2012 y 2012-2013.

1.34.2.2 Visita de selección (días -28 a 0)

Lactante preseleccionado que cumpla los siguientes criterios:

- Presenta un cuadro de bronquiolitis aguda
- Cumple todos los criterios de inclusión
- En la escala de valoración de la gravedad de Wood-Downes modificada por Ferres se clasifica como bronquiolitis aguda moderada
- No cumple ningún criterio de exclusión
- Los padres o tutores legales firman libremente el consentimiento informado
- Se realiza la anamnesis y exploración física y se procede a la extracción de la primera analítica (hemograma, bioquímica y gasometría) y la radiografía de tórax
- Se rellena la primera hoja de datos con las siguientes variables: Fecha de ingreso, número de historia clínica, iniciales de nombre y apellidos, fecha de nacimiento, antecedentes personales de interés, clínica de presentación del cuadro, escala de valoración de la gravedad, hallazgos exploratorios (fundamentalmente la auscultación respiratoria) y constantes vitales al ingreso

1.34.2.3 Visitas durante el periodo de tratamiento

Durante la fase de tratamiento se realizarán 2 visitas diarias por el personal médico, una a primera hora de la mañana, por el pediatra responsable del lactante donde decidirá sobre su tratamiento y evolución, y otra al final de la mañana, por un investigador colaborador que recabará datos específicos del estudio.

Visita 0:

- Una vez decidido el ingreso, el pediatra o residente de pediatría de guardia en Urgencias de Pediatría le realizará una historia clínica y una exploración física detallada, incluyendo la realización de la escala de gravedad (Wood-Downes modificada) para estimar la severidad de la bronquiolitis y su inclusión en el estudio si se clasifica como moderada.
- En la anamnesis se registrarán los factores de riesgo de presentar una bronquiolitis severa como son la edad menor de 12 semanas, el antecedente de prematuridad, enfermedad cardiopulmonar de base o presentar una inmunodeficiencia. Entre los antecedentes personales y familiares se preguntará específicamente por la presencia de atopia (dermatitis atópica, rinitis alérgica o asma), por episodios de sibilancias anteriores y por la existencia de fumadores en el domicilio habitual.
- En la exploración física se registrarán los signos de dificultad respiratoria como son la frecuencia respiratoria, tiraje, uso de musculatura accesoria,

cianosis o aleteo nasal y los hallazgos a la auscultación respiratoria como son las sibilancias espiratorias y/o inspiratorias, crepitantes, roncus, disminución del murmullo vesicular, hipoventilaciones o silencio auscultatorio así como si son simétricos o asimétricos. La frecuencia respiratoria será medida durante un periodo de un minuto, y la ausencia de taquipnea excluirá la infección de vías respiratorias bajas. Se preguntará y registraran los episodios de apnea.

- Las exploraciones complementarias que se realizarán al ingreso son hemograma, bioquímica con PCR, gasometría venosa, radiografía de tórax y un aspirado nasal para el test del VRS, como se hace de manera rutinaria en los pacientes de estas características.

- Según el grado de dificultad respiratoria y/o la tolerancia oral al ingreso, o durante el mismo, se pautarán tomas pequeñas y más frecuentes o bien, se mantendrán en dieta absoluta con fluidoterapia intravenosa según sus necesidades basales hasta que mejore la sintomatología respiratoria y pueda reanudarse la alimentación oral.

- Todos los pacientes recibirán las medidas generales recogidas en el protocolo diagnóstico-terapéutico conocido por todos los facultativos y que está disponible por escrito tanto en Urgencias de Pediatría como en Planta de Hospitalización. Además, todos los pediatras y residentes de la Unidad de Gestión Clínica de Pediatría disponen de una copia escrita del protocolo. Las medidas generales que incluyen el protocolo son: elevación de la cabecera de la cama 20º, oxigenoterapia en catéter o gafas nasales para mantener una saturación por encima del 94%, paracetamol si fiebre y lavados nasales con suero fisiológico antes de las tomas y antes de las nebulizaciones. Además se dejará por escrito en la hoja de tratamiento la recomendación de lavarse las manos y el fonendo antes y después de cada visita a un niño con bronquiolitis.
- A su ingreso, los pacientes serán monitorizados con un aparato de pulsioximetría continua transcutánea que permitirá detectar de forma precoz situaciones de hipoxia y regular, según protocolo, la FiO2 a administrar. Igualmente, se registrará el balance hídrico diario que permita el manejo correcto de los líquidos aportados.
- Los pacientes serán asignados de forma aleatorizada siguiendo una secuencia aleatoria generada por un ordenador, a uno de los dos siguientes grupos:

Grupo experimental: recibirá nebulizaciones de placebo más suero salino hipertónico al 3%.

Grupo control: recibirá nebulizaciones de adrenalina más suero salino hipertónico al 3%

- La generación de la lista de aleatorización correrá a cargo de la Unidad de Gestión Clínica de Farmacia
- El investigador principal dispondrá del listado de asignación al tratamiento en sobre cerrado, ante la eventual necesidad de romper el ciego por un acontecimiento adverso.

- El nebulizador utilizado es un nebulizador ultrasónico hospitalario Shinmed modelo Sw918 con una frecuencia ultrasónica de 1.7 Mhz. y un tamaño de nebulización de 1-5 micras.

- De inicio, todos los pacientes recibirán nebulizaciones cada 4 horas. Según la evolución clínica, las nebulizaciones se podrán administrar hasta un máximo de cada 3 horas si la evolución no es satisfactoria o bien se irán espaciando a cada 6 y posteriormente a cada 8 horas hasta el alta médica, si la evolución es satisfactoria.

Visita 1:

- Al día siguiente de su ingreso, el pediatra referente, previo lavado de manos y fonendo, le realizará una anamnesis dirigida a la evolución clínica percibida por los padres desde el ingreso (estado general, apetito y tolerancia, dificultad respiratoria, inquietud, fiebre, somnolencia y alivio con la medicación recibida) y se repetirá la exploración física, incluyendo la realización de la escala de Wood Downes modificada.
- En la exploración física se registrarán los signos de dificultad respiratoria como son la frecuencia respiratoria, tiraje, uso de musculatura accesoria, cianosis o aleteo nasal y los hallazgos a la auscultación respiratoria como son las sibilancias espiratorias y/o inspiratorias, crepitantes, roncus, disminución del murmullo vesicular, hipoventilaciones o silencio auscultatorio así como si son simétricos o asimétricos. La frecuencia respiratoria será medida durante un periodo de un minuto, y la ausencia de taquipnea excluirá la infección de vías respiratorias bajas. Se preguntará y registraran los episodios de apnea.
- Las exploraciones complementarias no se repetirán a menos que la evolución no haya sido satisfactoria.
- Según el grado de dificultad respiratoria y/o la tolerancia oral desde el ingreso, se continuará con tomas pequeñas y más frecuentes o bien, se mantendrá en dieta absoluta con fluidoterapia intravenosa según sus necesidades basales hasta que mejore la sintomatología respiratoria y pueda reanudarse la alimentación oral.
- Se comprobará que recibe las medidas generales recogidas en el protocolo diagnóstico-terapéutico de la bronquiolitis (elevación de la cabecera de la cama 20º, oxigenoterapia en catéter o gafas nasales para mantener una saturación por encima del 94%, paracetamol si fiebre y lavados nasales con suero fisiológico antes de las tomas y antes de las nebulizaciones).
- Se revisará el buen funcionamiento de la monitorización (pulsioximetría continua transcutánea) y se revisará las constantes tomadas por los distintos turnos de enfermería del día anterior (Frecuencia respiratoria, frecuencia cardiaca, diuresis y saturación de oxígeno)
- Se preguntará a padres y personal sanitario, expresamente, por los posibles efectos adversos percibidos
- Según evolución clínica y monitorización de constantes se decidirá espaciar, mantener o acortar el tiempo entre nebulizaciones.
- Si en cualquier momento durante la mañana o la guardia, el paciente empeorase clínicamente se le administrará una nebulización de rescate, previo lavado de fosas nasales con suero fisiológico y se le aumentará el aporte de oxígeno, si precisa. Si no hay mejoría, se repetirá la analítica y radiografía de tórax para ver la evolución con el día anterior y si se clasifica

como una bronquiolitis grave se contactará con la Unidad de Cuidados Intensivos Pediátricos, dándose por finalizado el estudio de este paciente.

Visita 2:

- Al segundo día de su ingreso, el pediatra referente, previo lavado de manos y fonendo, le realizará de nuevo una anamnesis dirigida a la evolución clínica percibida por los padres desde el ingreso (estado general, apetito y tolerancia, dificultad respiratoria, inquietud, fiebre, somnolencia y alivio con la medicación recibida) y se repetirá la exploración física, incluyendo la realización de la escala de Wood Downes modificada.
- En la exploración física se registrarán los signos de dificultad respiratoria como son la frecuencia respiratoria, tiraje, uso de musculatura accesoria, cianosis o aleteo nasal y los hallazgos a la auscultación respiratoria como son las sibilancias espiratorias y/o inspiratorias, crepitantes, roncus, disminución del murmullo vesicular, hipoventilaciones o silencio auscultatorio así como si son simétricos o asimétricos. La frecuencia respiratoria será medida durante un periodo de un minuto, y la ausencia de taquipnea excluirá la infección de vías respiratorias bajas. Se preguntará y registraran los episodios de apnea.
- Las exploraciones complementarias no se repetirán a menos que la evolución no haya sido satisfactoria.
- Según el grado de dificultad respiratoria y/o la tolerancia oral del día anterior, se continuará con tomas pequeñas y más frecuentes o bien, se mantendrá en dieta absoluta con fluidoterapia intravenosa según sus necesidades basales hasta que mejore la sintomatología respiratoria y pueda reanudarse la alimentación oral.
- Se comprobará que recibe las medidas generales recogidas en el protocolo diagnóstico-terapéutico de la bronquiolitis.
- Se revisará el buen funcionamiento de la monitorización (pulsioximetría continua transcutánea) y se revisará las constantes tomadas por los distintos turnos de enfermería del día anterior (Frecuencia respiratoria, frecuencia cardiaca, diuresis y saturación de oxígeno)
- Se preguntará a padres y personal sanitario, expresamente, por los posibles efectos adversos percibidos
- Según evolución clínica y monitorización de constantes se decidirá espaciar, mantener o acortar el tiempo entre nebulizaciones.
- Si en cualquier momento durante la mañana o la guardia, el paciente empeorase clínicamente se le administrará una nebulización de rescate, previo lavado de fosas nasales con suero fisiológico y se le aumentará el aporte de oxígeno, si precisa. Si no hay mejoría, se repetirá la analítica y radiografía de tórax para ver la evolución con el día anterior y si se clasifica como una bronquiolitis grave se contactará con la Unidad de Cuidados Intensivos Pediátricos, dándose por finalizado el estudio de este paciente.

Visita 3

- Al tercer día de su ingreso, el pediatra referente, previo lavado de manos y fonendo, le realizará de nuevo una anamnesis dirigida a la evolución clínica

percibida por los padres desde el ingreso (estado general, apetito y tolerancia, dificultad respiratoria, inquietud, fiebre, somnolencia y alivio con la medicación recibida) y se repetirá la exploración física, incluyendo la realización de la escala de Wood Downes modificada.

- En la exploración física se registrarán los signos de dificultad respiratoria como son la frecuencia respiratoria, tiraje, uso de musculatura accesoria, cianosis o aleteo nasal y los hallazgos a la auscultación respiratoria como son las sibilancias espiratorias y/o inspiratorias, crepitantes, roncus, disminución del murmullo vesicular, hipoventilaciones o silencio auscultatorio así como si son simétricos o asimétricos. La frecuencia respiratoria será medida durante un periodo de un minuto, y la ausencia de taquipnea excluirá la infección de vías respiratorias bajas. Se preguntará y registraran los episodios de apnea.
- Al tercer día se repetirá la realización de un hemograma, bioquímica y gasometría. La radiografía de tórax solo se repetirá en caso de mala evolución clínica.
- Según el grado de dificultad respiratoria y/o la tolerancia oral del día anterior, se continuará con tomas pequeñas y más frecuentes o bien, se mantendrá en dieta absoluta con fluidoterapia intravenosa según sus necesidades basales hasta que mejore la sintomatología respiratoria y pueda reanudarse la alimentación oral.
- Se comprobará que recibe las medidas generales recogidas en el protocolo diagnóstico-terapéutico de la bronquiolitis.
- Se revisará el buen funcionamiento de la monitorización (pulsioximetría continua transcutánea) y se revisará las constantes tomadas por los distintos turnos de enfermería del día anterior (Frecuencia respiratoria, frecuencia cardiaca, diuresis y saturación de oxígeno)
- Se preguntará a padres y personal sanitario, expresamente, por los posibles efectos adversos percibidos
- Según evolución clínica y monitorización de constantes se decidirá espaciar, mantener o acortar el tiempo entre nebulizaciones.
- Si en cualquier momento durante la mañana o la guardia, el paciente empeorase clínicamente se le administrará una nebulización de rescate, previo lavado de fosas nasales con suero fisiológico y se le aumentará el aporte de oxígeno, si precisa. Si no hay mejoría, se repetirá la analítica y radiografía de tórax para ver la evolución con el día anterior y si se clasifica como una bronquiolitis grave se contactará con la Unidad de Cuidados Intensivos Pediátricos, dándose por finalizado el estudio de este paciente.

Visita 4 hasta el alta del paciente:

- El resto de los días que permanezca ingresado hasta su alta, el pediatra referente, previo lavado de manos y fonendo, le realizará de nuevo una anamnesis dirigida a la evolución clínica percibida por los padres desde el ingreso (estado general, apetito y tolerancia, dificultad respiratoria, inquietud, fiebre, somnolencia y alivio con la medicación recibida) y se repetirá la exploración física, incluyendo la realización de la escala de Wood Downes modificada.

- En la exploración física se registrarán los signos de dificultad respiratoria como son la frecuencia respiratoria, tiraje, uso de musculatura accesoria, cianosis o aleteo nasal y los hallazgos a la auscultación respiratoria como son las sibilancias espiratorias y/o inspiratorias, crepitantes, roncus, disminución del murmullo vesicular, hipoventilaciones o silencio auscultatorio así como si son simétricos o asimétricos. La frecuencia respiratoria será medida durante un periodo de un minuto, y la ausencia de taquipnea excluirá la infección de vías respiratorias bajas. Se preguntará y registraran los episodios de apnea.
- No se repetirán la realización de pruebas complementarias salvo empeoramiento de su estado.
- Según el grado de dificultad respiratoria y/o la tolerancia oral del día anterior, se continuará con tomas pequeñas y más frecuentes o bien, se mantendrá en dieta absoluta con fluidoterapia intravenosa según sus necesidades basales hasta que mejore la sintomatología respiratoria y pueda reanudarse la alimentación oral.
- Se comprobará que recibe las medidas generales recogidas en el protocolo diagnóstico-terapéutico de la bronquiolitis.
- Se revisará el buen funcionamiento de la monitorización (pulsioximetría continua transcutánea) y se revisará las constantes tomadas por los distintos turnos de enfermería del día anterior (Frecuencia respiratoria, frecuencia cardiaca, diuresis y saturación de oxígeno)
- Se preguntará a padres y personal sanitario, expresamente, por los posibles efectos adversos percibidos.
- Según evolución clínica y monitorización de constantes se decidirá espaciar, mantener o acortar el tiempo entre nebulizaciones.
- Si en cualquier momento durante la mañana o la guardia, el paciente empeorase clínicamente se le administrará una nebulización de rescate, previo lavado de fosas nasales con suero fisiológico y se le aumentará el aporte de oxígeno, si precisa. Si no hay mejoría, se repetirá la analítica y radiografía de tórax para ver la evolución con el día anterior y si se clasifica como una bronquiolitis grave se contactará con la Unidad de Cuidados Intensivos Pediátricos, dándose por finalizado el estudio de este paciente.
- Los criterios de finalización del estudio son alcanzar una puntuación menor de 3 puntos en la escala de gravedad, saturación de oxígeno > 97% respirando aire ambiente, presentar adecuada tolerancia oral y la no necesidad de nebulizaciones.

1.34.2.4 VISITA DE SEGURIDAD

Un investigador colaborador realizará una visita diaria al paciente donde analizará su estado clínico y sus constantes vitales, tanto las recogidas en el dia anterior como las actuales, así como la posibilidad de efectos adversos.

1.35 Criterios de interrupción o finalización del ensayo

Criterios de finalización del estudio: tener una escala de gravedad ≤ 3 puntos, saturación de oxígeno > 97% respirando aire ambiente, adecuada tolerancia oral y la no necesidad de nebulizaciones.

Criterios de retirada prematura del estudio: los pacientes podrán ser retirados del estudio de forma prematura si tras su inclusión en el mismo, su estado empeora y se clasifica como bronquiolitis aguda grave. Igualmente, un paciente podrá ser retirado de forma prematura del estudio si, a juicio del investigador, presenta acontecimientos adversos graves, inesperados o clínicamente relevantes relacionados con los tratamientos en estudio.

1.36 Procedimientos para contabilizilar el medicamento en investigación

No es preciso realizar la contabilidad del fármaco para evaluar el cumplimiento terapéutico ya que los medicamentos en investigación serán administrados por el personal de enfermería de la planta de hospitalización. De forma diaria, el investigador principal o colaborador revisará las gráficas de enfermería para confirmar que el tratamiento ha sido administrado y recibido por el paciente.

Selección y retirada de sujetos

1.37 Criterios de inclusión de los sujetos

El criterio de inclusión será el de todo lactante menor de 24 meses sin patología de base y con necesidad de ingreso hospitalario por el diagnóstico clínico de bronquiolitis aguda, definida como el primer episodio de sibilancias asociada a un cuadro de infección de vías respiratorias altas durante la época epidémica, que su gravedad sea moderada y cuyos padres o tutores consientan de manera voluntaria participar en el estudio.

1.38 Criterios de exclusión de los sujetos

Los criterios de exclusión serán: enfermedad respiratoria crónica como anomalías congénitas, displasia broncopulmonar o la fibrosis quística, cardiopatía hemodinámicamente significativa, inmunodeficiencia, episodio anterior de sibilancias o diagnostico previo de asma, antecedentes de prematuridad menor de 37 semanas de gestación con edad corregida de menos de 6 semanas, haber recibido tratamiento previo o durante el ingreso con corticoides, enfermedad neuromuscular, pacientes con ventilación mecánica domiciliaria y pacientes con bronquiolitis grave que requieran ingreso en UCIP. También son criterios de exclusión cualquier otra patología grave que a juicio del investigador contraindique la participación en el estudio y la participación en estudios de investigación en los seis meses anteriores al momento del reclutamiento.

1.39 Criterios de retirada de los sujetos

Se podrá retirar aun paciente del estudio en las siguientes situaciones:

- Por revocación del consentimiento informado por parte de los padres o tutores legales
- Por evolución hacia bronquiolitis grave
- Por aparición de complicaciones, tipo sobreinfección bacteriana o neumotórax, que precisen un cambio de tratamiento
- Por presentar un efecto adverso grave, inesperado o clínicamente relevante
- Siempre que a criterio del investigador no sea recomendable mantener al paciente en el estudio
- Si cumple los criterios de finalización del estudio: escala de gravedad ≤ 3 puntos, saturación de oxígeno > 97% respirando aire ambiente, adecuada tolerancia oral y la no necesidad de nebulizaciones.

Tratamiento de los sujetos

1.40 Tratamiento de estudio

1.40.1 Descripción del tratamiento del estudio

Suero salino hipertónico al 3% nebulizado
Adrenalina nebulizada

Los grupos de tratamiento son dos:

1. Grupo experimental: recibirá nebulizaciones de suero salino hipertónico al 3%
2. Grupo control: recibirá nebulizaciones de suero salino hipertónico al 3%

más adrenalina.

1.40.2 Duración del tratamiento

Desde que cumple los criterios de inclusión al ingreso hasta que cumpla los criterios de alta del estudio (puntuación menor de 3 puntos en la escala de gravedad, saturación de oxígeno > 97% respirando aire ambiente, presentar adecuada tolerancia oral y la no necesidad de nebulizaciones), presente un efecto adverso relevante, los padres o tutores legales retiren el consentimiento, el médico responsable lo crea oportuno por su evolución o aparición de complicaciones o precise ingreso en UCIP por empeoramiento clínico o analítico (bronquiolitis grave)

1.40.3 Instrucciones de uso y administración del tratamiento

La medicación nebulizada consistirá en 3 ml de adrenalina 1:1000 más 5.5 ml de agua destilada más 1.5 ml de suero hipertónico al 20%, con un total de 10 ml, para el grupo control; y de 8.5 ml de agua destilada más 1.5 ml de suero hipertónico al 20% con un total de 10 ml, para el grupo de estudio. Ambas soluciones de nebulización tiene una concentración final de sodio del 3%.

La Unidad de Gestión Clínica de Farmacia, de acuerdo con el Art. 3.2 de la ORDEN SCO 256/2007, se encargará de preparar, envasar y etiquetar los medicamentos en investigación según la secuencia de aleatorización generada por ordenador identificándola con un código desconocido para médico y paciente. La medicación de ambos grupos tendrá el mismo volumen, peso, y características organolépticas.

La Unidad de Gestión Clínica de Farmacia solicitará la autorización previa de la AEMPS para la fabricación de medicamentos en investigación para acreditar que se cumplen las Normas de Correcta Fabricación y en particular el Anexo 13 de la Guía de NCF.

De inicio, todos los pacientes recibirán nebulizaciones cada 4 horas. Según la evolución clínica, las nebulizaciones se podrán administrar hasta un máximo de cada 3 horas si la evolución no es satisfactoria o bien se irán espaciando a cada 6 y posteriormente a cada 8 horas hasta el alta médica, si la evolución es satisfactoria. Los criterios de alta son alcanzar una puntuación menor de 3 puntos en la escala de gravedad, saturación de oxígeno > 97% respirando aire ambiente, presentar adecuada tolerancia oral y la no necesidad de nebulizaciones.

1.40.4 Modificación de los esquemas del tratamiento por acontecimientos adversos

En caso de afecto adverso se valorará su repercusión en el paciente y la relación con la medicación administrada. En caso de efectos adversos bien tolerados por el lactante se continuará con la administración de la medicación prescrita y se aumentará la monitorización de las constantes vitales (frecuencia cardiaca, frecuencia respiratoria, tensión arterial y saturación de oxígeno cada 3 horas)

Si se considera que el efecto adverso es mal tolerado por el paciente o cumple criterios de gravedad, se le retirará inmediatamente del estudio.

1.40.5 Procedimientos para monitorizar el cumplimiento del sujeto

En las gráficas de enfermería se comprobará que el medicamento nebulizado ha sido administrado según la pauta prescrita por el pediatra responsable del paciente.

1.40.6 Destrucción de la medicación del estudio

No es necesario, ya que al ser una medicación nebulizada, se consume y no precisa destrucción.

1.41 Medicamentos o tratamientos permitidos y no permitidos antes de y/o durante el ensayo

1.41.1 Tratamientos permitidos

Medidas de soporte: elevación de la cabecera de la cama 20º, oxigenoterapia en catéter o gafas nasales para mantener una saturación por encima del 94%, paracetamol si fiebre, lavados nasales con suero fisiológico antes de las tomas y antes de las nebulizaciones y fluidoterapia intravenosa si precisa (por mala tolerancia oral)

1.41.2 Tratamientos no permitidos

Medicamentos que no han demostrado beneficio en la evolución de la bronquiolitis:

- Corticoides por vía sistémica
- Corticoides por vía nebulizada
- Broncodilatadores orales
- Medicamentos que pueden interferir en la evaluación del estudio:
- Nebulización de otros broncodilatadores (salbutamol, bromuro de ipratropio)

1.41.3 Medicación de rescate

En caso de causar baja en el estudio por retirada del consentimiento informado, se le administrará las mismas medidas generales y las nebulizaciones serán de suero salino hipertónico al 3% más adrenalina (según protocolo de la Unidad Clínica de Gestión de Pediatría)

En caso de causar baja del estudio por presentarse un efecto adverso posiblemente relacionado con el suero salino hipertónico al 3% (por ejemplo broncoconstricción) se emplearán las mismas medidas generales y se administrarán nebulizaciones de suero salino fisiológico más adrenalina

Valoración de la eficacia

1.42 Especificación de los parámetros de eficacia

1.42.1 Parámetro principal de eficacia

Disminución de la estancia hospitalaria

1.42.2 Parámetros secundarios de eficacia

Reducción de la escala de gravedad

Mejoría en la evolución clínica

Mejoría de los parámetros gasométricos

Normalización de las constantes vitales

Disminución de la necesidad de ventilación mecánica

1.43 Los métodos y el calendario para la evaluación, registro y análisis de los parámetros de eficacia

El calendario de evaluación se detalla en el anexo 2.

Un investigador colaborador realizará una visita diaria para registrar la evolución clínica, la escala de gravedad, las constantes vitales, el número de nebulizaciones recibidas, el resultado analítico (si lo tiene), la presencia o ausencia de posibles eventos adversos y la conveniencia de continuar en el estudio.

así mismo, en colaboración con su pediatra referente, valoraran la evolución del paciente, y más específicamente la reducción de la escala de gravedad, la mejoría en la evolución clínica y de los parámetros gasométricos y la normalización de las constantes vitales. En el análisis estadísitco que se realizará cada 2 meses se valorará la disminución de la estancia hospitalaria y de la necesidad de ventilación mecânica.

Valoración de seguridad

1.44 Especificación de los parámetros de seguridad

Las evaluaciones de la seguridad incluirán:

Acontecimientos adversos, incluidos los AA de especial interés, como la taquicardia, taquipnea, hipertensión, broncoconstricción, palidez, sudoración y temblor
Constantes vitales: presión arterial, frecuencia cardíaca, frecuencia respiratoria y temperatura corporal
Datos de la exploración física (notificados como AA)
Parámetros analíticos habituales: bioquímica, hematología

1.45 Métodos para la evaluación, registro y análisis de los parámetros de seguridad

En cada visita, los investigadores evaluarán la aparición de acontecimientos adversos y dejarán constancia de los mismos tanto en la historia clínica del paciente como en el CRD

1.45.1 Acontecimiento adverso, definición

Es todo aquel efecto que se presente durante el tiempo que el paciente permanezca en el hospital con el diagnóstico de bronquilitis moderada aguda y haya recibido, al menos, una dosis del tratamiento.

1.45.2 Recogida y notificación de acontecimientos adversos

Es responsabilidad del investigador notificar todos los acontecimientos adversos en el CRD, tanto observados por él como notificados espontáneamente por el paciente participante en el estudio, independientemente de la relación con los tratamientos.

Todos los acontecimientos adversos se deben notificar durante todas las fases del estudio, y se debe realizar un seguimiento hasta su resolución, o hasta que se encuentre una explicación adecuada, aunque el paciente haya completado el tratamiento del estudio.

En todas las visitas, cada sujeto será interrogado sobre la presencia de nuevos acontecimientos adversos o sobre la evolución de los pre-existentes.

El periodo de notificación de acontecimientos adversos comienza tras la administración de la primera dosis de medicación del estudio (los acontecimientos adversos presentes en la primera visita se notificarán en la historia clínica basal, sólo la modificación de la gravedad o evolución será notificada como acontecimientos adversos en las siguientes visitas).

La notificación de acontecimientos adversos continuará hasta 12 horas de la última dosis administrada del tratamiento del estudio. También se registrarán los acontecimientos adversos que el investigador considere relacionados con el tratamiento del estudio, durante la fase de seguimiento de supervivencia.

Se recogerá la siguiente información relacionada con el acontecimiento adverso:

- Descripción.
- Duración.
- Resolución.
- Intensidad máxima.
- Relación causal con el/los tratamientos del estudio.
- Gravedad máxima.

Intensidad

La intensidad de los acontecimientos adversos se clasificará utilizando las criterios de terminología común para acontecimientos adversos del Nacional Cancer Institute, versión 4.0 (NCI-CTCAE) y se registrará de forma detallada, tal y como indique el CRD. Si se manifiesta un acontecimiento adverso que no esté incluido en el sistema de clasificación NCI-CTCAE, se utilizará la escala de 5 puntos que se detalla a continuación:

1. Leve: Malestar general, sin interrupción de la actividad diaria normal.
2. Moderado: Malestar general suficiente para reducir o afectar la actividad diaria normal.

3. Severo: Incapacidad para el trabajo o el desarrollo de la actividad diaria normal.
4. Amenazante para la vida o incapacitante: Representa una amenaza inmediata para la vida.
5. Muerte: Relacionada con el acontecimiento adverso.

Relación causal

En todos los acontecimientos adversos se establecerá la relación causal del acontecimiento con cada uno de los fármacos del estudio.

Existen tres categorías de causalidad:

- No relacionado: Definitivamente no relacionado con el fármaco. El acontecimiento se considera clara e indiscutiblemente relacionado con causas externas (enfermedad, entorno, etc.).
- Relacionado: Incluye aquellos acontecimientos que están remotamente, posiblemente, probablemente o definitivamente relacionados con el fármaco.
- Relación desconocida.

Todos los acontecimientos adversos clasificados por el investigador como relacionados con el fármaco, se clasificarán como reacciones adversas a los fármacos.

Gravedad

Todos los acontecimientos se clasificarán en graves o no graves, y esta clasificación determinará el procedimiento de notificación del acontecimiento.

Será considerado un acontecimiento adverso grave, cualquier acontecimiento adverso o reacción adversa al fármaco que, a cualquier dosis:

- Produzca la muerte (la muerte es un resultado, no un acontecimiento, por lo que no se podrá considerar muerte como una identificación del acontecimiento adverso).
- Amenace la vida (no se incluyen en esta categoría aquellos acontecimientos que hipotéticamente podrían haber causado la muerte de haber sido más graves).
- Haga necesaria la hospitalización o la prolongación de ésta (se considera hospitalización un ingreso no planeado, de al menos una noche).
- Produzca invalidez o incapacidad permanente o importante.
- Dé lugar a una anomalía o malformación congénita.
- A efectos de su notificación, se tratarán también como graves aquellas sospechas de acontecimientos adversos que se consideren clínicamente relevantes aunque no cumplan los criterios anteriores.

Notificación de alteraciones de laboratorio

Los resultados de las pruebas de laboratorio se registrarán en el apartado correspondiente del CRD.

Los valores anómalos de las pruebas de laboratorio se registrarán como acontecimientos adversos siempre que el acontecimiento adverso:

- Esté asociado con una afección clínica del paciente que requiera tratamiento.
- Provoque modificación del tratamiento concomitante.
- Provoque una interrupción o retraso en el tratamiento del estudio, o la modificación de la dosis de uno o varios de los fármacos del estudio.
- Se trate de un acontecimiento adverso grave.
- El paciente sea definitivamente retirado del estudio debido al valor anómalo de la prueba.

<u>Notificación de acontecimientos adversos graves</u>

Cualquier acontecimiento adverso o anomalía de laboratorio que sea grave y ocurra durante el desarrollo del estudio, independientemente del tratamiento recibido por el paciente, deberá notificarse mediante el envío del formulario de acontecimientos graves al monitor del estudio,., dentro de las 24 horas siguientes a su conocimiento y lo comunicará al promotor-investigador principal

Los acontecimientos adversos, graves, inesperados y posiblemente relacionados con la medicación del ensayo deberán comunicarse también a los CEIC implicados y a las autoridades sanitarias

La notificación se adherirá a la definición y requerimientos de las guías para la gestión de datos de seguridad, definiciones y estándares para la notificación expeditiva de las guías ICH Tema E2.

La información mínima a incluir en la notificación inicial del acontecimiento adverso grave incluye: identificación del paciente, identificación del acontecimiento adverso, fecha de inicio, motivo por el que se considera grave, relación causal con la medicación del estudio y nombre de la persona que originariamente notifica el acontecimiento.

Toda la información adicional sobre un acontecimiento adverso grave previamente notificado, deberá también notificarse en el formulario de notificación de acontecimientos adversos graves, en las 24 horas posteriores a su conocimiento.

La notificación inmediata del acontecimiento adverso en el formulario será adicional a la recogida de estos datos en el CRD.

1.46 SEGUIMIENTO DE LOS SUJETOS DESPUÉS DE LOS ACONTECIMIENTOS ADVERSOS

<u>Acontecimientos adversos graves o amenazantes para la vida, no relacionados:</u>

- Monitorización estrecha de sus constantes y valores anómalos
- Administración de tratamiento si precisa
- Valoración de la necesidad de ingresar en la Unidad de Cuidados Críticos Pediátricos

<u>Acontecimientos adversos graves o amenazantes para la vida, relacionados:</u>

- Suspender la medicación y finalización del estudio en ese paciente

- Monitorización estrecha de sus constantes y valores anómalos
- Administración de tratamiento si precisa
- Valoración de la necesidad de ingresar en la Unidad de Cuidados Críticos Pediátricos

ESTADÍSTICA

1.47 DESCRIPCIÓN DE LOS MÉTODOS ESTADÍSTICOS QUE SE USARÁN, INCLUYENDO EL CALENDARIO DE TODOS LOS ANÁLISIS INTERMEDIOS PLANIFICADOS

Para los análisis intermedios y final se considerará la estancia hospitalaria como variable dependiente principal y como variables dependientes secundarias la evolución de la escala de gravedad, constantes vitales, evolución clínica, presencia de efectos secundarios y necesidad de ventilación mecánica.

Se realizarán análisis descriptivos de variables cuantitativas mediante media, mediana, desviación típica y rango, y frecuencia y porcentaje para las variables cualitativas.

Cada variable categórica de interés se examinará en ambos grupos mediante la prueba de "Ji-cuadrado" de Mantel-Henszel, con la corrección de Yates o prueba exacta de Fischer si precisa.

Se comprobará la normalidad de la distribución de las variables cuantitativas estudiadas mediante el test de Kolmogorov-Smirnov y se realizarán comparaciones de medias, entre el grupo control y experimental, con el test de T de Student y prueba de Levene de homogeneidad de varianzas en variables paramétricas y U de Mann-Whitney en caso de variables no paramétricas. (* optativo: El análisis de la varianza de una vía, ANOVA se realizará en caso de comparación de los datos entre los diferentes centros implicados). Para examinar el cambio de variables cuantitativas, se realizará para cada grupo de tratamiento separadamente, un análisis antes-después con T-test de muestras pareadas. La diferencia o caída de la variable entre ambos grupos, se examinará usando el T-test para muestras independientes.

El Plan de Análisis estadístico propuesto contempla análisis intermedios cada 3 meses y un análisis final a los 3 años del inicio del ensayo

1.48 NÚMERO PREVISTO DE SUJETOS QUE SE INCLUIRÁN

Dado que el ensayo se basa en demostrar no la superioridad, sino la equivalencia en la variable dependiente principal entre el grupo control y experimental, el cálculo del tamaño muestral se basó en el establecimiento de unos límites o márgenes de bioequivalencia (por encima y por debajo de la eficacia media del producto de referencia), que se estableció como la mayor diferencia en la estancia hospitalaria aceptable y que debía ser menor que la diferencia observada en estudios revisados de superioridad (2 días). De esta forma, y estableciendo que la diferencia mínima aceptable en la estancia hospitalaria entre ambos grupos debía ser de un día, fijando el riesgo α o probabilidad del error tipo I en un 5% y el riesgo β o probabilidad del error tipo II en un 5%, con una razón de muestreo 1:1 entre el grupo control y experimental, resultó un tamaño muestral de 104 pacientes por grupo de estudio, con un total de 208 pacientes. Esta estimación del tamaño muestral es orientativa y necesaria para establecer el cronograma y el tiempo minimo que se requerirán para alcanzar resultados concluyentes.

1.49 NIVEL DE SIGNIFICACIÓN QUE SE UTILIZARÁ

El nivel de significación previamente establecido será del 95% ($p < 0.05$), para la mayoría de las variables dependientes (principal y algunas secundarias) y en el caso de análisis del cambio con múltiples comparaciones, dado que se trata de la monitorización de datos que hace que la

probabilidad de encontrar un resultado estadísticamente significativo vaya incrementándose, se fijará en el 99% ($p<0,001$ en las comparaciones iniciales aumentando progresivamente hasta un α global del 5% siguiendo la regla de Peto-Haybittle).

1.50 Criterios para la finalización del ensayo

Los análisis intermedios o interinos ayudarán a decidir si el ensayo ha de terminar con anterioridad en base a la presencia de efectos secundarios, a la mayor necesidad de ventilación mecánica o a la peor evolución clínica en el grupo experimental. Esto también podrá ocurrir si de forma precoz se demuestra un claro beneficio de la terapia experimental, pues los análisis intermedios se relacionarán con la eficacia del tratamiento que se valora.

En estos análisis se monitorizarán datos de las tasas de reclutamiento por centros, estadísticas descriptivas de la población de estudio, cumplimiento por grupos, datos de seguridad y de eficacia (especialmente para los efectos primarios, y también para las variables secundarias). Para ello se valorará la creación de un Comité de Monitorización de Datos, multidisciplinar, sin conflicto de intereses y que serán los únicos que conozcan los resultados de los análisis intermedios sobre la eficacia de los tratamientos.

En principio, se fijará el principios estadístico para detener la progresión del estudio de un valor p muy pequeño al principio del ensayo.
Se utilizarán pruebas estadísticas de dos colas.

Se valorará realizar junto al análisis secuencial de grupos, la detención aleatoria utilizando los datos observados y los posibles datos no observados, asumiendo la hipótesis nula para el resto del ensayo.

1.51 Procedimiento utilizado para contabilizar los datos perdidos, no utilizados y erróneos

Se calculará la proporción de pérdidas durante el seguimiento, con descripción de los retiros y abandonos. Si las pérdidas ocurren por igual en los grupos experimental y control y si son inferiores al 20% es menos probable que se afecte la validez interna: la distribución del resto de factores de riesgo conocidos en los sujetos perdidos, se analizarán de forma exploratoria para orientarnos de la magnitud del problema.

Se realizará un análisis por intención de tratar, para controlar el sesgo de confusión que podría producirse por el incumplimiento o desviación de lo establecido según protocolo, en el que cada individuo permanece en el grupo al que fue asignado inicialmente, con independencia del tratamiento que finalmente haya recibido y de si lo completó o no (a partir de la población total aleatorizada o al menos de aquellos con alguna evaluación prevista en el estudio realizada). Dado que se describe que este tipo de análisis puede no resultar del todo beneficioso en los estudios de equivalencia, se comprobará si los resultados de eficacia coinciden con los del análisis de eficacia biológica donde se comparan los sujetos que completaron el tratamiento al que fueron asignados, lo cual fortalecerá el análisis.

Acceso Datos/Documentos Fuente

El archivo del estudio: Sólo tendrá acceso a él el investigador principal.

El cuaderno de recogida de datos: Todos los investigadores colaboradores dispondrán de fotocopias del cuaderno de recogida de datos para su debida cumplimentación diaria. Una vez finalizado el estudio de cada paciente serán entregados al investigador principal para su archivo.

La historia clínica. Se realiza al ingreso del paciente, y posteriormente se escribe la evolución clínica y analítica de forma diaria

Control y Garantía de Calidad

1.52 Monitorización del estudio

La monitorización del estudio se realizará según los PNT del investigador principal y según las recomendaciones establecidas en la ICH Topic E6. Guideline for Good Clinical Practice. CPMP/ICH/135/95.

1.53 Auditorias e inspecciones

El investigador/institución debe facilitar el acceso a los datos/documentos originales para la monitorización del ensayo, auditorías, revisiones del CEIC e inspecciones reguladoras. En cumplimiento con todas las regulaciones aplicables, se exige que el investigador y el centro permitan a los representantes autorizados del promotor del estudio, de la(s) agencia(s) reguladora(s) y al CEIC el acceso directo a la revisión de los registros médicos originales de los pacientes para la verificación de los procedimientos y datos relacionados con el estudio. Este acceso directo incluye la exploración, análisis, verificación y reproducción de cualquier registro o informe que sea importante para la evaluación del estudio. El investigador está obligado a informar y obtener el consentimiento del sujeto para permitir que los representantes designados tengan acceso a sus registros relacionados con el estudio sin quebrantar la confidencialidad del sujeto. La verificación de los datos del CRD se debe realizar por inspección directa de los documentos originales.

Ética

1.54 Legislación / Declaración de Helsinki / ICH

El presente estudio clínico se realizará de acuerdo con el protocolo, los principios establecidos en la versión revisada actual de la Declaración de Helsinki (Seúl, 2008) y de acuerdo con los requisitos reguladores aplicables, en particular las Normas armonizadas tripartitas ICH para las buenas prácticas clínicas 1996 y el Real Decreto de Ensayos Clínicos 223/2004, por el que se regulan los ensayos clínicos con medicamentos en España, y que incorpora en su totalidad el ordenamiento de la Directiva europea 2001/20/CE, relativa a las disposiciones de los estados miembros sobre la aplicación de buenas prácticas clínicas en la realización de ensayos clínicos de medicamentos de uso humano.

El investigador consiente, cuando firma el protocolo, en adherirse a las instrucciones y procedimientos descritos en ellos y de esta manera, seguir los principios de las buenas prácticas clínicas que ellos implican.

El estudio no empezará hasta tener la aprobación escrita del CEIC de referencia, así como de la AEMPS.

Cualquier enmienda que cambie la relación beneficio-riesgo para el paciente deberá ser, una vez firmada por el promotor, sometida a la evaluación de los CEICs y de la AEMPS para su aprobación. Los investigadores del estudio también serán informados y darán su aprobación escrita de la misma.

1.55 Información a los participantes del estudio

Manejo de los Datos y Archivo de los Registros

1.56 Confidencialidad

Con el fin de garantizar la confidencialidad de los datos del estudio, sólo tendrán acceso a los mismos el investigador y su equipo de colaboradores, el monitor, el representante del promotor, el CEIC y las autoridades sanitarias pertinentes.

El contenido de los cuadernos de recogida de datos, así como los documentos generados durante el estudio y la base de datos electrónica, serán protegidos de usos no permitidos por personas ajenas a la investigación y, por tanto, serán considerados estrictamente confidenciales y no serán revelados a terceros excepto a los especificados en el apartado anterior. El tratamiento de los datos de carácter personal requeridos en este estudio se rige por la Ley Orgánica 15/1999, de 13 de diciembre, de protección de datos de carácter personal.

Todo material, información (oral o escrita), documentación no publicada que sea facilitada a los investigadores, incluyendo este protocolo, los cuadernos de recogida de datos y el manual del investigador, deben considerarse como propiedad del promotor.

Es obligación del investigador considerar como confidencial y asegurar en todo momento la confidencialidad de los documentos y resultados generados durante el transcurso del ensayo, exceptuando los que la legislación defina como divulgables.

Los datos y/o material del estudio no podrán ser divulgados, en parte o en su totalidad, por el investigador o sus colaboradores a ninguna persona no autorizada, sin el consentimiento formal previo y por escrito del promotor.

Financiación y Seguros

Tal y como exigen la legislación vigente, y en particular el Real Decreto 223/04, el promotor contratará una póliza de seguro que cubra las responsabilidades del promotor, del investigador principal y sus colaboradores, y del hospital o centro donde se lleve a cabo el ensayo clínico, antes posibles daños y perjuicios que afecten a la salud del paciente durante la realización del estudio y en el año siguiente a la terminación del mismo, salvo prueba en contrario.

Política de Publicación

Las publicaciones derivadas del estudio se regirán por las previsiones del artículo 38 del Real Decreto 223/2004, del 6 de febrero, por el que se regulan los ensayos clínicos con medicamentos y conforme al cual, constituye una

obligación del promotor, la publicación de los resultados del estudio, tanto positivos como negativos, en revistas científicas, y con mención al Comité Ético de Investigación Clínica que aprobó el estudio, así como la fuente de financiación, garantizándose en todo momento, el anonimato de los pacientes participantes en el mismo.

Toda la información relacionada con el estudio es considerada confidencial y propiedad del promotor-investigador hasta su publicación. Una vez que el estudio haya finalizado y se haya realizado el informe estadístico, el equipo investigador elaborará el informe final del estudio, que se presentará a los CEICs, la AEMPS y las autoridades sanitarias que así lo soliciten. Este informe final será la base para la elaboración de los manuscritos que se deseen publicar en revistas médicas.

ANEXO 1. Bibliografía

1. Subcommittee on diagnosis and management of bronchiolitis. Diagnosis and management of bronchiolitis. Pediatrics 2006; 118 (4): 1774-1793
2. García martín fj, moreno pérez d. Bronquiolitis. Protocolos diagnósticos-terapéuticos.
3. Centers for disease control and prevention. Respiratory syncytial virus. Www.cdc.gov/ncidod/dvrd/respiratory/rsvfeat.htm (accessed 2008 june 23)
4. Hall cb, walsh ee, long ce, schnabel kc. Inmunity to and frecuency of reinfection with respiratory syncytial virus. J infect dis 1991; 163: 693-698.
5. Willson df, horn sd, hendley jo, smout r, gassaway j. Effect of practice variation on resource utilization in infants for viral lower respiratory illness. Pediatrics 2001; 108: 851-855.
6. Behrendt ce, decker md, burch dj, watson ph. International variation in the management of infants hospitalized with respiratory syncytial virus. International rsv study group. Eur j pediatr 1998; 157: 215-220
7. Patel h, platt rw, pekeles gs, ducharme fm. A randomized, controlled trial of the effectiveness of nebulized therapy with epinephrine compared with albuterol and saline in infants hospitalized for acute viral bronchiolitis. J pediatri 2002; 141 (6): 818-24
8. Wainwright c, altamirano l, chehey m, cheney j, barber s, price d, moloney s, et al. A multicenter, randomized, double-blind, controlled trial of nebulized epinephrine in infants with acute bronchiolitis. N england j med 2003; 349 (1): 27-35
9. Hartling l, wiebe n, russell k, patel h, klassen tp. A meta-analysis of randomized controlled trials evaluating the efficacy of epinephrine for the treatment of acute viral bronchiolitis. Arch pediatr adolesc med 2003; 157 (10): 957-64
10. Hartling l, wiebe n, rusell k, patel h, klassen tp. Epinephrine for bronchiolitis. Cochrane database syst rev 2004.
11. Langley jm, smith mb, leblanc jc, joudrey h, ojah cr, pianosi p. Racemic epinephrine compared to salbutamol in hospitalized young children with bronchiolitis; a randomized controlled clinical trial. Bmc pediatr 2005; 5 (1): 7
12. Mandelberg a, tal g, witzling m, someck e, houri s, balin a, priel ie. Nebulized 3% hypertonic saline solution treatment in hospitalized infants with viral bronchiolitis. Chest 2003; 123 (2): 481-7
13. Tal g, cesar k, oron a, houri s, ballin a. Hypertonic saline/epinephrine treatment in hospitalized infants with viral bronchiolitis reduces hospitalization stay: 2 years experience. Imaj 2006; 8 (3): 169-73
14. Kuzik b, qadhi s, kent s, flavin mp, hopman w, hotte s, gander s. Nebulized hypertonic saline in the treatment of viral bronchiolitis in infants. J pediatr 2007; 151 (9): 266-70
15. Linjie z, mendoza-sassi r, wainwright c, klassen tp. Solución salina hipertónica nebulizada para la bronquiolitis aguda en recién nacidos. Cochrane database syst rev 2007.
16. Balaguer santamaría a, buñuel alvarez jc, gonzalez de dios j. El suero salino hipertónico nebulizado puede disminuir la duración del ingreso hospitalario en lactantes con bronquiolitis aguda. Evid pediatr 2009; 5: 5
17. Zimment i. Respiratory pharmacology and therapeutics. Philadelphia: wb saunders, 1978.

18. Robinson m, hemming a, regnis j, wong a, baley d, bautotvich gj et al. Effect of increasing doses of hypertonic saline on mucociliary clearance in patients with cystic fibrosis. Thorax 1997; 52: 900-903.
19. Aussouline g, leibson v, danon a. Stimulation of prostaglandin output from rat stomach by hypertonic olution. European journal of pharmacology 1977; 44: 271-273
20. Cataldo d, foidant jm, lau l, bartsch p, djukanovic r, louis r. Induced sputum: comparison between isotonic and hypertonic saline solution in patients with asthma. Chest 2001; 120: 1815-1821
21. Suri r, marchall lj, wallis c, mecalfe c, shute jk, bush a. Safety and use of sputum induction in children with cystic fibrosis. Pediatr pulmonol 2003; 35: 309-313
22. Rodwell lt, anderson sd. Airway responsiveness to hyperosmolar saline challenge in cystic fibrosis: a pilot study. Pediatr pulmonol 1996; 21: 282-289
23. Palomeque a., martín jmª, cambra fj, pons m, jordan i, esteban e, et al. Guía terapéutica en intensivos pediátricos, 4ª edición. Pgn. 123

ANEXO 2. CALENDARIO DE EVALUACIONES

Procedimientos	Pre-selección	Selección	Fase de tratamiento	Visita segurida
Diagnostico de bronquiolitis aguda	X			
Cumple los criterios de inclusión		X		
No cumple criterios de exclusión		X		
Firma consentimiento informado		X		
Analítica general		X		
Radiografía de tórax		X		
Historia clínica y exploración		X		
Posición elevada del cabecero			X	
Lavados nasales con suero			X	
Aspiración de secreciones			X	
Hidratación oral o iv			X	
Oxigenoterapia			X	
Fraccionamiento de tomas			X	

Nebulización de la medicación	X
Controles constantes vitales	X
Medición diuresis	X
Escala de gravedad diaria	X
Monitorización efectos adversos	X
Supervivencia	

ANEXO 3. **CALENDARIO DE SEGURIDAD**

**Procedimientos**	**Pre-selección**	**Selección**	**Fase de tratamiento**	**se[illegible]**
Patología de base	X			
Alteración analítica de base	X	X		
Radiografía de tórax	X	X		
Escala de gravedad		X	**X**	
Evolución clínica			X	
Episodios de broncoconstricción			**X**	
Taquicardia			**X**	
Taquipnea			X	
Hipertensión arterial			X	
Sudoración			X	
Temblor			X	
Palidez			X	
Comprobar administración medicación			X	
Otros efectos adversos inesperados			X	

ANEXO 4. **MODELO CONSENTIMIENTO INFORMADO**

HOJA DE INFORMACIÓN AL REPRESENTANTE LEGAL/ PADRES DEL PARTICIPANTE EN UN ESTUDIO DE INVESTIGACIÓN CLÍNICA

Título del estudio: ENSAYO CLÍNICO ALEATORIZADO, DOBLE CIEGO, CONTROLADO Y PARALELO PARA COMPARAR LA EFICACIA DE LA NEBULIZACIÓN DE SUERO SALINO HIPERTÓNICO AL 3% CON ADRENALINA FRENTE A NEBULIZACIÓN DE SUERO SALINO HIPERTÓNICO AL 3% EN NIÑOS HOSPITALIZADOS CON BRONQUIOLITIS AGUDA MODERADA

Investigador principal (Nombre, servicio, forma de localizarle): Jose Carlos Flores González, Intensivista Pediátrico.

Centro: Hospital Universitario Puerta del Mar de Cádiz

1.56.1.1.1.1 INTRODUCCION

Se le invita a participar en un estudio que ha sido aprobado por la Subcomisión de Investigación/Subcomisión de Ética del Hospital Universitarios Puerta del Mar

Por favor, lea esta hoja informativa con atención. El Dr./Dra: ____ le aclarará las dudas que le puedan surgir.

1.56.1.1.1.2 PARTICIPACIÓN VOLUNTARIA

Su participación en este estudio es voluntaria y usted puede anular su decisión y retirar el consentimiento en cualquier momento sin que por ello se altere su relación con el médico ni se produzca perjuicio en su tratamiento o en la atención que usted pueda necesitar.

1.56.1.1.1.3 DESCRIPCIÓN GENERAL DEL ESTUDIO

El objetivo de este estudio consiste en comparar 2 tratamientos aprobados para la enfermedad de su hijo o hija. No hay ningún tratamiento placebo, ambos han demostrado su eficacia en disminuir el tiempo de hospitalización de los lactantes con bronquiolitis, pero intentamos comprobar si uno aporta más beneficio que otro. Además de uno de estos tratamientos, recibirá el resto de cuidados y medicación protocolizada para la bronquiolitis. No existe ninguna medicación curativa para la bronquiolitis, solo disponemos de medicamentos que mejoran su evolución.

Los dos tratamientos han sido previamente utilizados en los lactantes con bronquiolitis y han demostrado su utilidad, sin efectos secundarios. Los niños que necesitaron ingreso en UCIP y ventilación mecánica fueron por la mala evolución de la bronquiolitis y no por el tratamiento. Hasta la actualidad no se han descrito efectos secundarios de estos tratamientos. Se realizará una revisión periódica de los datos obtenidos para comparar su eficacia y seguridad.

Durante su ingreso recibirá la visita diaria de su pediatra referente que decidirá libremente sobre la evolución y tratamiento de su hija o hijo, y una visita del investigador, también diaria, en la cual registrará todas las variables para comprobar la conveniencia de continuar en el estudio.

La forma de actuación médica y el protocolo del tratamiento de la bronquiolitis no cambia con este estudio. Recibirá el mismo tratamiento que muchos niños con la misma enfermedad que su hijo o hija. Si decide no incorporarse al estudio o retirarse una vez empezado, el tratamiento que utilizaremos será similar a los del estudio ya que no existen en la actualidad otras alternativas terapéuticas.

Hay un porcentaje de niños con bronquiolitis que evolucionan mal independientemente del tratamiento utilizado, y necesitan ser atendidos en la UCI pediátrica y recibir ventilación mecánica. Si esto ocurriese su hijo o hija será excluido del estudio en el acto.

Para este estudio no se le realizará ninguna prueba invasiva a su hijo o hija que no esté indicada por la enfermedad. Este estudio no implica ninguna medida extraordinaria. Tras el alta usted no tendrá que acudir al hospital para revisiones. La duración del estudio es el

tiempo que permanezca su hija o hijo hospitalizado. En este estudio participarán más de 100 pacientes.

1.56.1.1.1.4 BENEFICIOS Y RIESGOS DERIVADOS DE SU PARTICIPACIÓN EN EL ESTUDIO

Se espera que su participación en este estudio le proporcione los siguientes beneficios: Disminución de la estancia hospitalaria, reducción de la gravedad de la enfermedad de su hijo o hija, y menor necesidad de ingreso en la UCI pediátrica. Además, la información que se obtenga servirá para ampliar el conocimiento científico sobre la bronquiolitis, una enfermedad muy frecuente y que implica numerosos ingresos en recién nacidos y lactantes.

Los resultados de este estudio podrían así ayudarnos a seleccionar el mejor tratamiento para la bronquiolitis, evitando otros de menor utilidad o que pudieran tener más efectos secundarios.

1.56.1.1.1.5 COMPENSACIÓN ECONÓMICA

Su participación en el estudio no supondrá ningún gasto para usted.

1.56.1.1.1.6 CONFIDENCIALIDAD

Sus datos serán tratados con la más absoluta confidencialidad según lo dispuesto en la Ley Orgánica 15/1999, de 13 de diciembre de Protección de Datos de Carácter Personal. De acuerdo a lo que establece la legislación mencionada, usted puede ejercer los derechos de acceso, modificación, oposición y cancelación de datos, para lo cual deberá dirigirse al investigador responsable del ensayo, Dr Jose Carlos Flores González (956002255).

Los datos recogidos para el estudio estarán identificados mediante un código y sólo el investigador principal/colaboradores podrán relacionar dichos datos con usted y con su historia clínica.

Si se publican los resultados del estudio, sus datos personales no serán publicados y su identidad permanecerá anónima.

1.56.1.1.1.7 FINANCIACIÓN

Este estudio está financiado por el Ministerio de Sanidad y Consumo

1.56.1.1.1.8 RETIRADA DEL CONSENTIMIENTO

Usted puede retirar su consentimiento en cualquier momento sin tener que dar explicaciones. Si usted no desea participar más en el estudio y usted lo quiere así, todas sus muestras identificables serán destruidas para evitar la realización de un nuevo análisis.

También debe saber que puede ser excluido del ensayo si (el promotor) o los investigadores del estudio lo consideran oportuno.

Usted tiene derecho a estar informado de cualquier proyecto de nuevos análisis del material identificable retenido no previsto en este estudio. El investigador podría tener que pedirle un nuevo consentimiento que usted podría rechazar.

Antes de firmar, lea detenidamente el documento, haga todas las preguntas que considere oportunas, y si lo desea, consúltelo con todas las personas que considere necesario. En caso de duda debe dirigirse al Dr. Jose Carlos Flores González

Firma del representante legal: **Firma del Investigador:**

Nombre: **Nombre:**

Fecha: **Fecha:**

MODELO DE CONSENTIMIENTO INFORMADO POR ESCRITO

Título del estudio: ENSAYO CLÍNICO ALEATORIZADO, DOBLE CIEGO, CONTROLADO Y PARALELO PARA COMPARAR LA EFICACIA DE LA NEBULIZACIÓN DE SUERO SALINO HIPERTÓNICO AL 3% CON ADRENALINA FRENTE A NEBULIZACIÓN DE SUERO SALINO HIPERTÓNICO AL 3% EN NIÑOS HOSPITALIZADOS CON BRONQUIOLITIS AGUDA MODERADA

Promotor: Jose Carlos Flores González

1.56.1.2 YO (NOMBRE Y APELLIDOS)

____

He leído la hoja de información que se me ha entregado.

He podido hacer preguntas sobre el estudio.

He recibido suficiente información sobre el estudio.

He hablado con:

____

(nombre del investigador)

Comprendo que mi participación es voluntaria.

Comprendo que puedo retirarme del estudio:

1º Cuando quiera

2º Sin tener que dar explicaciones.

3º Sin que esto repercuta en mis cuidados médicos.

Presto libremente mi conformidad para participar en el estudio.

FECHA: FIRMA DEL REPRESENTANTE LEGAL

MODELO DE CONSENTIMIENTO ORAL ANTE TESTIGOS

Título del estudio: ENSAYO CLÍNICO ALEATORIZADO, DOBLE CIEGO, CONTROLADO Y PARALELO PARA COMPARAR LA EFICACIA DE LA NEBULIZACIÓN DE SUERO SALINO HIPERTÓNICO AL 3% CON ADRENALINA FRENTE A NEBULIZACIÓN DE SUERO SALINO HIPERTÓNICO AL 3% EN NIÑOS HOSPITALIZADOS CON BRONQUIOLITIS AGUDA MODERADA

Promotor: J Carlos Flores González

Yo (nombre y apellidos) ___________________________________ declaro bajo mi responsabilidad que ___________________________________ (nombre del participante en el ensayo)

Ha recibido la hoja de información sobre el estudio.

Ha podido hacer preguntas sobre el estudio.

Ha recibido suficiente información sobre el estudio.

Ha sido informado por:

. ___________________________________

(nombre del investigador)

Comprende que su participación es voluntaria.

Comprende que puede retirarse del estudio:

1º Cuando quiera

2º Sin tener que dar explicaciones.

3º Sin que esto repercuta en sus cuidados médicos.

Y ha expresado libremente su conformidad para participar en el estudio.

FECHA: FIRMA DEL TESTIGO

MODELO DE CONSENTIMIENTO DEL REPRESENTANTE

Título del estudio: ENSAYO CLÍNICO ALEATORIZADO, DOBLE CIEGO, CONTROLADO Y PARALELO PARA COMPARAR LA EFICACIA DE LA NEBULIZACIÓN DE SUERO SALINO HIPERTÓNICO AL 3% CON ADRENALINA FRENTE A NEBULIZACIÓN DE SUERO SALINO HIPERTÓNICO AL 3% EN NIÑOS HOSPITALIZADOS CON BRONQUIOLITIS AGUDA MODERADA

Promotor: Jose Carlos Flores González

Yo (nombre y apellidos) ____________________________________ en calidad de ____________________________________ (relación con el participante) de ____ (nombre del participante en el ensayo)

He leído la hoja de información sobre el estudio.

He podido hacer preguntas sobre el estudio.

He recibido respuestas satisfactorias a mis preguntas

He recibido suficiente información sobre el estudio.

He hablado con:

. _______________________________________

(nombre del investigador)

Comprendo que la participación es voluntaria.

Comprende que puede retirarse del estudio:

1º Cuando quiera

2º Sin tener que dar explicaciones.

3º Sin que esto repercuta en sus cuidados médicos.

En mi presencia se ha dado a ____ (nombre del participante en el ensayo) toda la información pertinente adaptada a su nivel de entendimiento y está de acuerdo en participar.

Y presto mi conformidad con que ____ (nombre del participante en el ensayo) participe en este estudio.

FECHA: FIRMA DEL REPRESENTANTE

ANEXO V CONTENIDO DEL FORMULARIO DE CI SEGÚN LAS NORMAS DE BPC CPMP/ ICH/ 135/ 95

La discusión del consentimiento informado, el formulario de consentimiento informado y cualquier otra información escrita facilitada a los sujetos deben incluir las explicaciones siguientes:

1. Que el ensayo supone investigación.

2. El propósito del ensayo.

3. El tratamiento del ensayo y la probabilidad de asignación aleatoria para cada tratamiento.

4. Los procedimientos a seguir en el ensayo, incluyendo todos los procedimientos invasivos.

5. Las responsabilidades del sujeto.

6. Aquellos aspectos del ensayo que son experimentales.

7. Los riesgos o inconveniencias razonablemente previsibles para el sujeto y, cuando sea pertinente para el embrión, feto o niño.

8. Los beneficios razonablemente esperados. Cuando no se pretende ningún beneficio clínico para el sujeto debe estar informado de ello.

9. Los procedimientos alternativos o tratamientos que puedan existir para el sujeto y sus riesgos y beneficios potenciales importantes.

10. La indemnización y/o tratamiento disponible para el sujeto en el caso de daños relacionados con el ensayo.

11. El prorrateo anticipado de pago, si lo hay, al sujeto participante en el ensayo.

12. Los gastos anticipados, si los hay, al sujeto participante en el ensayo.

13. Que la participación del sujeto en el ensayo es voluntaria y que el sujeto puede rechazar su participación o abandonar un ensayo sin penalización o pérdida de los beneficios a los que hubiera tenido derecho de otro modo.

14. Que el monitor (es), auditor (es), el CEIC y las autoridades reguladoras tendrán garantizado el libre acceso a la historia clínica original del sujeto para la verificación de los procedimientos y/o datos del ensayo clínico sin violar la confidencialidad del sujeto, dentro de los permitido por las leyes y regulaciones pertinentes, y que, firmando el formulario de consentimiento informado, el sujeto o el representante legalmente aceptable del sujeto están autorizando tal acceso.

15. Que los documentos que identifiquen al sujeto serán confidenciales y, dentro de lo permitido por las leyes y regulaciones pertinentes, no estarán a disposición pública. Si se publican los resultados del ensayo, la identidad del sujeto será confidencial.

16. Que el sujeto o el representante legalmente aceptable del sujeto será informado en todo momento si se dispone de nueva información que pueda ser relevante para la decisión del sujeto de continuar participando en el ensayo.

17. La persona o personas con quién contactar para obtener información adicional acerca del ensayo y de los derechos de los sujetos del ensayo y con quien contactar en caso de perjuicios relacionados con el ensayo.

18. Las circunstancias previsibles y/o las razones bajo las cuales puede finalizar la participación del sujeto en el ensayo.

19. La duración prevista de la participación del sujeto en el ensayo.

20. El número aproximado de sujetos implicados en el ensayo.
