# Supplementary figures and images for: Epinephrine Improves the Efficacy of Nebulized Hypertonic Saline in Moderate Bronchiolitis: A Randomised Clinical Trial
Source: PLoS One. 2015 Nov 17;10(11):e0142847. doi: 10.1371/journal.pone.0142847 (PMC4648584; doi:10.1371/journal.pone.0142847)

## CONSORT 2010 Flow Diagram

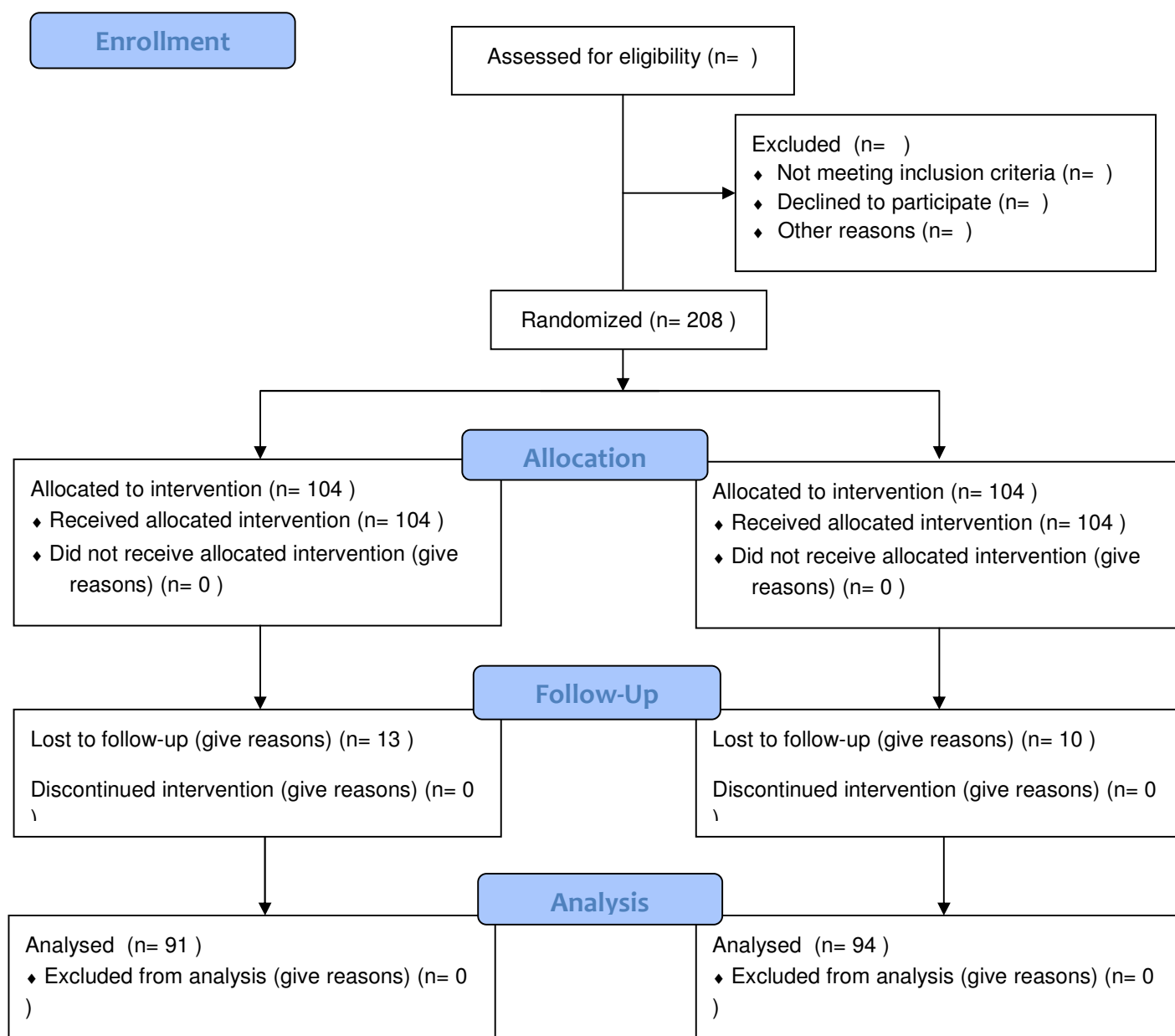

Supplement: S3 File — (PDF) [file pone.0142847.s003.pdf]
